# A Low-Rank Method for Characterizing High-Level Neural Computations

**DOI:** 10.3389/fncom.2017.00068

**Published:** 2017-07-31

**Authors:** Joel T. Kaardal, Frédéric E. Theunissen, Tatyana O. Sharpee

**Affiliations:** ^1^Computational Neurobiology Laboratory and Crick-Jacobs Center for Theoretical and Computational Biology, Salk Institute for Biological Studies La Jolla, CA, United States; ^2^Center for Theoretical Biological Physics, University of California, San Diego La Jolla, CA, United States; ^3^Department of Psychology, University of California, Berkeley Berkeley, CA, United States

**Keywords:** neural coding, auditory cortex, computational neuroscience, receptive fields, dimensionality reduction

## Abstract

The signal transformations that take place in high-level sensory regions of the brain remain enigmatic because of the many nonlinear transformations that separate responses of these neurons from the input stimuli. One would like to have dimensionality reduction methods that can describe responses of such neurons in terms of operations on a large but still manageable set of relevant input features. A number of methods have been developed for this purpose, but often these methods rely on the expansion of the input space to capture as many relevant stimulus components as statistically possible. This expansion leads to a lower effective sampling thereby reducing the accuracy of the estimated components. Alternatively, so-called low-rank methods explicitly search for a small number of components in the hope of achieving higher estimation accuracy. Even with these methods, however, noise in the neural responses can force the models to estimate more components than necessary, again reducing the methods' accuracy. Here we describe how a flexible regularization procedure, together with an explicit rank constraint, can strongly improve the estimation accuracy compared to previous methods suitable for characterizing neural responses to natural stimuli. Applying the proposed low-rank method to responses of auditory neurons in the songbird brain, we find multiple relevant components making up the receptive field for each neuron and characterize their computations in terms of logical OR and AND computations. The results highlight potential differences in how invariances are constructed in visual and auditory systems.

## 1. Introduction

Signal processing in neurobiological systems involves multiple nonlinear transformations applied to multidimensional inputs. Characterizing these transformations is difficult but essential to understanding the neural basis of perception. For example, neurons from successive stages of sensory systems represent inputs in terms of increasingly complex combinations of stimulus features (Felleman and Van Essen, 1991; King and Nelken, 2009). Although a number of statistical tools have been developed to analyze responses of sensory neurons, analysis of high-level sensory neurons remains a challenge because of two interrelated factors. First, to signal the presence of certain objects or events, high-level sensory neurons perform sophisticated computations that are based on multidimensional transformations of the inputs, which together form the *receptive field* of the neuron. Second, high-level neurons are unresponsive to noise stimuli and usually require structured stimuli reflective of the natural sensory environment. However, even when presented with natural stimuli, the specific combinations of inputs necessary to elicit responses of a given neuron do not occur frequently. As a result, current statistical methods fail to recover receptive fields for many high-level neurons due to a lack of sufficient sampling of the stimulus/response distribution relative to the number of model parameters. Therefore, to systematically probe high-level responses we need statistical methods that (i) estimate multidimensional transformations of the inputs, (ii) account for the biases in natural stimuli or other strongly correlated distributions, and (iii) are resistant to overfitting. Here we describe a practical method that satisfies these criteria and apply the method to gain new insights into the structure of receptive fields of high-level auditory neurons from the zebra finch auditory forebrain.

Present dimensionality reduction methods for recovering receptive fields of sensory neurons can be roughly divided into linear and quadratic methods. Linear methods attempt to reconstruct components of a neuron's receptive field by correlating the neural response to a set of features composed of stimulus components, *s*_*i*_. Examples of these methods include the spike-triggered average (STA), maximally informative dimensions (MID), and first-order maximum noise entropy (MNE) methods (Sharpee et al., 2004; Bialek and de Ruyter van Steveninck, 2005; Schwartz et al., 2006; Fitzgerald et al., 2011b). With MID being a notable exception, many of these linear methods are only capable of recovering a single component of the receptive field. The necessity of characterizing multiple components of receptive fields has led to the development of quadratic methods where the feature space is expanded quadratically to include all pairwise products, *s*_*i*_*s*_*j*_, between the components of a *D*-dimensional stimulus vector, **s** (Schwartz et al., 2006; Fitzgerald et al., 2011b; Park and Pillow, 2011; Rajan and Bialek, 2013). Generally speaking, such quadratic methods construct a weight matrix, **J**, that captures correlations between a neuron's responses and the quadratic feature space. The relevant subspace of stimulus space that spans the receptive field is recovered by diagonalizing **J**.

Methods designed to recover this relevant subspace can be susceptible to bias when the model is constructed based on incorrect assumptions. For instance, the spike-triggered covariance (STC) method (the quadratic analog of the STA method) assumes that the stimulus components are drawn from a Gaussian white noise distribution (Bialek and de Ruyter van Steveninck, 2005). When STC is applied to other stimulus distributions such as natural stimuli, the receptive field estimation is susceptible to bias and often leads to a poor reconstruction of the receptive field components. In response to this short-coming of the STC method, the MID and MNE methods were developed to minimize bias using principles from information theory. In the case of the MID method, components are found that maximize the mutual information between the response and stimuli independent of the nonlinear function relating stimuli to responses, also called the *nonlinearity* (Sharpee et al., 2004). The MNE method instead invokes the principle of maximum entropy to construct a nonlinearity that maximizes the noise entropy, *H*_noise_(*y*|**s**), between the response, *y*, and stimulus distribution subject to constraints on the response-weighted moments of the stimulus space; e.g., 〈*y*〉, 〈*y***s**〉, and 〈*y***ss**^T^〉 (Jaynes, 2003; Fitzgerald et al., 2011a,b). In order to minimize bias in the receptive field estimate, *H*_noise_ is maximized subject to only these data-dependent constraints.

Our proposed method builds on the second-order MNE model for the probability of a binary response given a set of stimuli (Fitzgerald et al., 2011a). This model is a logistic function of a linear combination of inputs in the expanded feature space (truncated here to second-order):

(1) $$\begin{array}{l} P(y=1|\text{s})=\frac{1}{1+e^{-z(\text{s})}},\;z(\text{s})=a+{\text{h}}^{\text{T}}\text{s}+{\text{s}}^{\text{T}}\text{Js} \end{array}$$

where unknown weights *a*, **h**, and **J** are determined by minimizing the negative log-likelihood. The order of the moments used in constructing the MNE model correspond to the order of the polynomial that appears in the argument, *z*(**s**). Including the *n*th-order constraint in the MNE model leads to an additional *D*^*n*^ weights that must be estimated. The MNE model is truncated to second-order to facilitate the reconstruction of multi-component receptive fields while avoiding the curse of dimensionality that appears when including constraints on the model from higher-order moments. At the same time, the second-order model is sufficient to describe contributions from multiple components that excite and suppress the neurons' response (Schwartz et al., 2006); one can approximate selectivity for higher-than-second-order features through combinations of pairwise constraints (Perrinet and Bednar, 2015). Other advantages of this approach are that (i) it works with arbitrary, including natural, stimuli, and (ii) the optimization is convex, converging swiftly to a global optimum. The disadvantage of this model is that, for a *D*-dimensional stimulus vector **s**, one needs to determine 1 + *D* + *D*(*D* + 1)/2 parameters of which only 1 + *D* + *rD* parameters will be ultimately used to specify *r* components obtained by diagonalizing the *D* × *D* matrix **J**. Note that an arbitrary antisymmetric matrix may be added to **J** without changing the output of the nonlinearity, *P*, and it is therefore sufficient, but not necessary, to optimize an MNE model with **J** constrained to be symmetric where only *D*(*D* + 1)/2 elements of **J** need to be optimized. However, this constraint was not part of the original optimization procedure (Fitzgerald et al., 2011a,b). Below we show that adding a constraint that ensures symmetric **J** improves the estimation accuracy in our proposed model.

For currently available datasets, the over-expansion of the stimulus space can be a severe limitation leading to overfitting of quadratic models. To resolve this issue, we designed low-rank MNE models with an explicit rank constraint where a rank *r* matrix **J** is modeled as a product of two low-rank *D* × *r* matrices, **J** = **UV**^T^ (Burer and Monteiro, 2003; Bach et al., 2008; Rajan and Bialek, 2013; Haeffele et al., 2014). For models where *r* ≪ *D*, this bilinear factorization leads to a substantial reduction in the number of parameters that are necessary to estimate. Furthermore, as is the case with many optimization methods, one can improve the robustness of estimation to noise from limited sampling through regularization that penalizes the magnitude of certain model parameters. Since we seek low-rank representations of **J**, we choose to invoke *nuclear-norm* (or trace-norm) regularization to penalize **J** based on the sparsity of its eigenvalue spectrum which, in addition to improved estimation, has the advantage of allowing us the flexibility to set *r* as an upper bound on the rank of **J** while applying regularization to further reduce the rank of **J** (Fazel, 2002; Fazel et al., 2003; Recht et al., 2010). We apply the low-rank MNE method to recover receptive field components from recordings of neurons in regions field L and the caudal mesopallium (CM) of the zebra finch auditory forebrain subject to auditory stimulation. Our results provide novel insights into the structure of multicomponent auditory receptive fields and suggest important differences between object-level respresentations in the auditory and visual cortex.

## 2. Results

### 2.1. Mathematical approach for low-rank characterization of neural feature selectivity

#### 2.1.1. Problem set-up

An optimal low-rank MNE model is one that minimizes the negative log-likelihood function with respect to the weights *a*, **h**, **U**, and **V**. The mean negative log-likelihood is:

(2) $$\begin{array}{l} L(a,\text{h},\text{U},\text{V})=-\frac{1}{N}\sum_{t}\left[y_{t}\log(P_{t})+(1-y_{t})\log(1-P_{t})\right] \end{array}$$

where *P*_*t*_ is introduced as a short-hand to represent the nonlinearity, *P*(*y* = 1|**s**_*t*_), *N* is the number of samples, and *y*_*t*_ ∈ [0, 1] is the response to *t*th sample of the stimulus space, **s**_*t*_.

Additional structure can be imposed on the weights by adding a penalty function to the mean negative log-likelihood (Equation 2). Nuclear-norm regularization is defined as the sum of absolute values of the eigenvalue spectrum (i.e., $\underset{k}{\sum }|\sigma_{k}|$ where σ_*k*_ is the *k*th eigenvalue of **J**). When applied as a penalty function, the nuclear-norm increases the sparsity of **J**'s eigenvalue spectrum (Fazel, 2002; Fazel et al., 2003; Recht et al., 2010). Because matrix **J** can possess both positive and negative eigenvalues, the nuclear-norm does not simply equal its trace. While in principle one can compute the nuclear-norm of **J** by diagonalization it is in practice more efficient to embed **J** within a larger positive semidefinite matrix, (Fazel, 2002; Fazel et al., 2003):

(3) $$\begin{array}{l} \text{Q}{\text{Q}}^{\text{T}}=\left[\begin{array}{c} \text{U}\\ \text{V} \end{array}\right]\left[\begin{array}{cc} {\text{U}}^{\text{T}}, & {\text{V}}^{\text{T}} \end{array}\right]=\left[\begin{array}{cc} \text{U}{\text{U}}^{\text{T}}, & \text{J}\\ {\text{J}}^{\text{T}}, & \text{V}{\text{V}}^{\text{T}} \end{array}\right] \end{array}$$

and instead take the trace over **QQ**^T^:

(4) $$\begin{array}{l} \ell_{*}(\text{Q})=\frac{1}{2}\sum_{k=1}^{r}\epsilon_{k}{\left\|{\text{Q}}_{•,k}\right\|}_{2}^{2}=\frac{1}{2}\sum_{k=1}^{r}\epsilon_{k}\left({\left\|{\text{U}}_{•,k}\right\|}_{2}^{2}+{\left\|{\text{V}}_{•,k}\right\|}_{2}^{2}\right) \end{array}$$

where ‖·‖_2_ is the ℓ_2_-norm and **Q**_•,*k*_, **U**_•,*k*_, and **V**_•,*k*_ refer to the *k*th column of each matrix. Here, ϵ_*k*_ ≥ 0 is a regularization parameter which is a hyperparameter that controls the strength of the nuclear-norm penalty. Regularizing over this semidefinite embedding penalizes the rank of **UU**^T^ and **VV**^T^. This leads to a penalization of the rank of **J** by proxy since rank(**J**) ≤ min(rank(**U**), rank(**V**)) (Fazel, 2002; Fazel et al., 2003; Cabral, 2013) shown in the following.

*Proof*. Since **U** spans the same range space as **UU**^T^ and **V** spans the same range space as **VV**^T^, it can be shown that regularizing over the trace of **QQ**^T^ is an effective strategy for regularizing the rank of **J** by showing that **J** has zero projection into the null space of **U** and **V**. The null space operators of **U** and **V** are defined as $P_{N}\left(\text{U}\right)=\text{I}-\text{U}{\text{U}}^{†}$ and $P_{N}\left(\text{V}\right)=\text{I}-\text{V}{\text{V}}^{†}$ where † indicates a generalized matrix inverse. Projecting **J** onto $P_{N}\left(\text{U}\right)$ yields $P_{N}\left(\text{U}\right)\text{J}=\text{J}-\text{U}{\text{U}}^{†}\text{U}{\text{V}}^{\text{T}}=\text{J}-\text{U}{\text{V}}^{\text{T}}=\text{0}$. Similarly, $\text{J}P_{N}\left(\text{V}\right)=\text{0}$. Therefore, the range space of **J** is a subset of the range spaces of **U** and **V** where rank(**J**) ≤ min(rank(**U**), rank(**V**)) and penalizing Tr(**QQ**^T^) (where Tr(·) is the trace) is an effective surrogate to the nuclear-norm of **J** for penalizing the rank of **J**.

This surrogate regularization readily works with gradient based methods for optimization, whereas diagonalization of **J** does not. Unlike typical implementations of the nuclear-norm that use only a single regularization parameter (i.e., ϵ_*k*_ = ϵ for all *k*), we found that assigning a unique regularization parameter for each of the *r* columns of **Q** led to substantial improvement in the characterization of **J**. A single regularization parameter has the tendency to eliminate insignificant components at the expense of degrading the quality of the significant high variance components. Using multiple regularization parameters allows us to eliminate the insignificant components while avoiding degradation of the significant components.

While the bilinear factorization of **J** into **U** and **V** sets an upper bound of *r* on the rank of **J**, there is a subtle inconsistency in how the rank behaves caused by the symmetry of the problem. In the present formulation of the optimization problem, **J** can be nonsymmetric and therefore possess an undesirable complex eigenvalue spectrum. This issue cannot simply be solved by symmetrizing $\text{J}\leftarrow {\text{J}}_{\text{sym}}=\frac{1}{2}\left(\text{J}+{\text{J}}^{\text{T}}\right)$ post-optimization. While symmetrizing would provide a real eigenvalue spectrum, this symmetrization procedure can have the unintended consequence of increasing the rank of **J** up to 2*r*. This can be a problem because there are generally not enough variables provided by the *D* × *r* matrices **U** and **V** to fit a rank 2*r* matrix. We resolved this inconsistency by requiring that **U** and **V** satisfy:

(5) $$\begin{array}{l} \text{U}{\text{V}}^{\text{T}}=\text{V}{\text{U}}^{\text{T}}\Rightarrow \text{J}={\text{J}}^{\text{T}}, \end{array}$$

with proof that this guarantees the rank of **J** is invariant to symmetrization provided in the following.

*Proof*. The symmetry constraint (Equation 5) is a sufficient condition to guarantee rank(**J**_sym_) ≤ *r* since $\mathrm{rank}\left({\text{J}}_{\text{sym}}\right)=\mathrm{rank}\left(\text{J}+{\text{J}}^{\text{T}}\right)=\mathrm{rank}\left(2\text{J}\right)\leq \min\left(\mathrm{rank}\left(\text{U}\right),\mathrm{rank}\left(\text{V}\right)\right)\leq r$.

From a practical point of view, the bilinear formulation of the symmetry constraints (Equation 5) is potentially problematic since its Jacobian can be rank-deficient and it introduces *D*(*D* − 1)/2 unique constraints which can lead to an overly large number of constraint equations to satisfy. The difficulty in applying constraints with rank-deficient Jacobian is that such constraints can fail to satisfy the Karush-Kuhn-Tucker (KKT) conditions (Prop 1 in the Appendix) when a local minimizer lies on the boundary of the feasible region. Since we require the application of equality constraints to enforce invariance of the rank of **J** to symmetrization, any feasible local minimum lies on the boundary of the feasible region. Consequently, we would like to formulate an optimization problem for low-rank MNE that will generally satisfy the KKT conditions at a local minimizer. A safe choice (see the discussion following Prop 1) is to replace the bilinear formulation with a set of *rD* linear equality constraints:

(6) $$\begin{array}{l} {\text{w}}_{k}={\text{U}}_{•,k}+\pi_{k}{\text{V}}_{•,k}={\text{A}}_{k,k}{\text{Q}}_{•,k}=0\text{ for all }k, \end{array}$$

where π_*k*_ ∈ {−1, 1} for the *k*th column of **U** and **V** and

(7) $$\begin{array}{l} {\text{A}}_{k,k}=\left[\begin{array}{cc} \text{I}, & \pi_{k}\text{I} \end{array}\right] \end{array}$$

is the *D* × 2*D* dimensional Jacobian matrix of **w**_*k*_ with respect to **Q**_•,*k*_. For a brief summary of alternative constraints that satisfy rank(**J**_sym_) ≤ *r*, see the Section 5.1 in the Appendix.

Putting this all together, the low-rank MNE method is a nonlinear program of the form:

(8) $$\begin{array}{l} \underset{a,\text{h},\text{Q}}{\min}f(a,\text{h},\text{Q})=\underset{a,\text{h},\text{Q}}{\min}L(a,\text{h},\text{Q})+\ell_{*}(\text{Q})\\ \text{subject to }{\text{w}}_{k}=0\text{ for all }k. \end{array}$$

This problem can be transformed from a constrained to “unconstrained” optimization via the Lagrangian method:

(9) $$\begin{array}{l} L(a,\text{h},\text{Q},\Lambda )=f(a,\text{h},\text{Q})-\sum_{k=1}^{r}\Lambda_{•,k}^{\text{T}}{\text{w}}_{k} \end{array}$$

where **Λ** is a *D* × *r* matrix of unconstrained Lagrange multipliers and:

(10) $$\begin{array}{l} f(a,\text{h},\text{Q})=L(a,\text{h},\text{Q})+\ell_{*}(\text{Q}). \end{array}$$

is the objective function. Alternatively, one may directly substitute **V**_•,*k*_ = −π_*k*_**U**_•,*k*_ into *f* for an equivalent unconstrained problem. Once a solution is found to Equation (8), relevant quadratic components of **J** are identified by diagonalizing **J**_sym_:

(11) $$\begin{array}{l} {\text{J}}_{\text{sym}}=\frac{1}{2}\left(\text{J}+{\text{J}}^{\text{T}}\right)=\Omega \Sigma \Omega^{\text{T}}, \end{array}$$

where **Ω** is a *D* × *D* matrix with columns forming an orthonormal basis and **Σ** is a *D* × *D* diagonal matrix where the Σ_*k,k*_ element corresponds to the variance of the **Ω**_•,*k*_ basis vector. Those columns of **Ω** with nonzero variance span the subspace of stimulus space relevant to a response (Fitzgerald et al., 2011a). Note that, in theory, a solution to Equation (8) should yield **J**_sym_ = **J** with maximum rank *r*. In practice, this is dependent on the desired precision to which the constraints are satisfied and at what variance the eigenvalues are defined to be approximately zero. If an investigator employs an eigenvalue solver that is more precise than the constraint satisfaction, diagonalizing **J** may result in a complex eigenvalue spectrum with small imaginary parts. Diagonalizing **J**_sym_ instead via Equation (11) eliminates these small imaginary components and will admit at most *r* eigenvalues with variance above the desired precision.

Unlike the full-rank MNE optimization, the low-rank MNE optimization is a nonconvex problem (see the discussion surrounding Prop 2 in the Appendix). This nonconvexity is caused by the bilinear factorization of **J** in the negative log-likelihood term of the cost function, *f*. The nuclear-norm penalty, on the other hand, is convex since ϵ_*k*_ ≥ 0 for all *k*. Due to this property of the nuclear-norm, it is possible to show that there is a regularization domain where any solution to the low-rank MNE problem is globally optimal (Burer and Monteiro, 2003; Bach et al., 2008; Haeffele et al., 2014). Specifically, if all ϵ_*k*_ are greater than or equal to the magnitude of the largest variance eigenvalue of the *D* × *D* gradient matrix **∇**_**J**_*L* (where **∇**_**J**_ is the gradient operator with respect to **J**) evaluated at a solution, then the weights *a*, **h**, **U**, and **V** are globally optimal solutions of the low-rank MNE problem. Conversely, if any ϵ_*k*_ is less than the magnitude of the largest variance eigenvalue of **∇**_**J**_*L*, then a solution to the low-rank MNE problem is not guaranteed to be globally optimal and belongs to the locally optimal domain. For proof of this, see the Sections 5.2 and 5.3 in the Appendix.

When the rank of the ground truth of matrix **J** is low-rank, solutions of the low-rank MNE problem in the globally optimal domain can be a good approximation to the ground truth of **J**. This approximate solution can be attractive due to its certifiable global optimality and can be helpful when *D* is practically too large to fit with the full-rank MNE method or to find compressed solutions for **J** of rank less than the ground truth. In some cases, however, it is possible that solutions that lie in the locally optimal domain better reconstruct the ground truth of **J** compared to solutions in the globally optimal domain. In the following sections, we detail optimization algorithms that may be used to find solutions in either the locally or globally optimal domains of the low-rank MNE problem.

#### 2.1.2. Optimizing the weights

To find a feasible local minimizer of the low-rank MNE problem (Equation 8) for given set of nuclear-norm regularization parameters, a line search interior-point method designed to find local minima of nonlinear, nonconvex programming problems based on Ch. 19 of *Numerical Optimization* by Nocedal and Wright (2006) is used. The interior-point method iteratively searches for a local minimum of the low-rank MNE minimization problem (Equation 8) by recursively solving:

(12) $$\begin{array}{l} \underset{H}{\underset{︸}{\left[\begin{array}{cc} \nabla_{\text{xx}}^{2}L, & {\text{A}}^{\text{T}}\\ \text{A}, & 0 \end{array}\right]}}\left[\begin{array}{c} {\text{p}}_{\text{x}}\\ -{\text{p}}_{\Lambda } \end{array}\right]=-\underset{\text{KKT}}{\underset{︸}{\left[\begin{array}{c} \nabla_{\text{x}}L\\ \text{w} \end{array}\right]}} \end{array}$$

for the weight and Lagrange multiplier update directions, **p**_**x**_ and **p**_**Λ**_, respectively, where a weight vector ${\text{x}}^{\text{T}}=\left[a,\;{\text{h}}^{\text{T}},\;{\text{Q}}_{•,1}^{\text{T}},\ldots \;,{\text{Q}}_{•,r}^{\text{T}}\right]$ is defined. The matrix **A** is the full Jacobian matrix of the constraints and **w** is a concatenation of the equality constraints (i.e., ${\text{w}}^{\text{T}}=\left[{\text{w}}_{1}^{\text{T}},\ldots \;,{\text{w}}_{r}^{\text{T}}\right]$). The matrix labeled H will be referred to as the constrained Hessian and the vector on the right-hand-side contains the KKT conditions (Prop 1 in the Appendix). For nonconvex problems, it can be useful to employ an optimization method that reduces the chances of converging to a saddle point of *f*. The implementation of the interior-point algorithm from Nocedal and Wright (2006) has the advantage of circumventing saddle points by adding a (1 + *D* + 2*rD*) × (1 + *D* + 2*rD*) positive diagonal shift matrix, δ**I** where δ > 0, to the Hessian of the Lagrangian, $\nabla_{\text{x}\text{x}}^{2}L+\delta \text{I}$, to maintain proper *matrix inertia* of the constrained Hessian. The matrix inertia is specified by the number of positive eigenvalues, *m*, the number of negative eigenvalues, *n*, and the number of eigenvalues equal to zero, *l*, of the constrained Hessian. To prevent convergence of the interior-point method to a saddle point of *f*, we maintain a matrix inertia of *m* = 1 + *D* + 2*rD* (the number of rows/columns of $\nabla_{\text{x}\text{x}}^{2}L$), *n* = *rD* (the number of constraints), and *l* = 0. If the constrained Hessian does not meet this condition, the inertia is enforced by adjusting δ until this condition is satisfied.

The trouble with using this interior-point method to solve Equation (8) is that the size of matrix H is (1 + *D* + 3*rD*) × (1 + *D* + 3*rD*) which can be prohibitively large for typical memory constraints and lead to substantial time spent solving the linear system (Equation 12). Some alternative approaches are to use quasi-Newton methods such as the Limited-memory Broyden-Fletcher-Goldfarb-Shanno (L-BFGS) algorithm (Nocedal and Wright, 2006) or gradient-only heuristics like stochastic gradient descent (Bottou, 2010). Another option is to divide the weights into blocks and perform block coordinate descent using constrained block Hessians (Wright, 2015). We chose the latter to better exploit the structure of the regularization function (Equation 4).

The block coordinate descent algorithm cyclically solves the subproblems:

(13) $$\begin{array}{l} \text{block }k\text{ subproblem}:\;\{\begin{array}{c} \underset{a,\text{h},{\text{Q}}_{•,k}}{\min}f\left(a,\text{h},\text{Q}\right)\\ \text{ subject to }{\text{w}}_{k}={\text{A}}_{k,k}{\text{Q}}_{•,k}=0 \end{array} \end{array}$$

until the KKT conditions (Prop 1 in the Appendix) and second-order sufficient conditions (Prop 2 in the Appendix) are satisfied. The block coordinate descent is performed by cyclically minimizing the cost function with respect to the *k*th block of weights ${\text{x}}_{k}^{\text{T}}=\left[a,{\text{h}}^{\text{T}},{\text{Q}}_{•,k}^{\text{T}}\right]$ using the interior-point algorithm described above to recursively solve:

(14) $$\begin{array}{l} \underset{H_{k}}{\underset{︸}{\left[\begin{array}{cc} \nabla_{{\text{x}}_{k}{\text{x}}_{k}}^{2}L, & {\text{A}}^{(k)\text{T}}\\ {\text{A}}^{\left(k\right)}, & 0 \end{array}\right]}}\left[\begin{array}{c} {\text{p}}_{{\text{x}}_{k}}\\ -{\text{p}}_{\Lambda_{k}} \end{array}\right]=-\left[\begin{array}{c} \nabla_{{\text{x}}_{k}}L\\ {\text{w}}_{k} \end{array}\right] \end{array}$$

while holding the remaining **Q**_•,*j*_ (*j* ≠ *k*) fixed. The new indexing on the Jacobian ${\text{A}}^{\left(k\right)\text{T}}=\nabla_{{\text{x}}_{k}}{\text{w}}_{k}^{\text{T}}$ is the Jacobian of the *k*th block constraints and is a *D* × (1 + 3*D*) matrix. Proof that the block coordinate descent algorithm converges to a feasible local minimizer of the low-rank MNE problem (Equation 8) appears in Section 5.4 of the Appendix.

#### 2.1.3. Hyperparameter optimization

Now we turn to the procedure for setting the nuclear-norm regularization parameters. In the globally optimal domain (Prop 4 in the Appendix), the goal is to use nuclear-norm regularization to find a globally optimal solution that approximates a solution to the unregularized problem where all ϵ_*k*_ = 0. Therefore, it makes sense to make the regularized and unregularized problems as similar as possible by using the minimal amount of regularization necessary to reach the globally optimal domain. To do so, one can optimize each block of the block coordinate descent such that ϵ_*k*_ is approximately equal to the magnitude of the largest variance eigenvalue of **∇**_**J**_*L*, which will be defined as λ_*L*_. A simple algorithm for achieving this is: (i) optimize **x**_*k*_, then (ii) increase ϵ_*k*_ if ϵ_*k*_ < λ_*L*_ or decrease ϵ_*k*_ if ϵ_*k*_ > λ_*L*_, and then repeat steps i and ii until ϵ_*k*_ ≈ λ_*L*_ for each block (see Algorithm 1 for a pseudocode implementation). By contrast, in the locally optimal regularization domain we instead adjust ϵ_*k*_ to find the model that best generalizes to novel data in a cross-validation set. This approach to hyperparameter optimization is in common use in modern machine learning applications (Bergstra et al., 2011; Bergstra and Bengio, 2012).

**Algorithm 1:** Low-rank MNE block coordinate descent algorithm (globally optimal domain)

1:	**inputs:** maximum rank *r*, paired data samples (**s**_*t*_, *y*_*t*_) for all *t*, initial guess for weights *a*, **h**, **U**, and **V**, set π_*k*_ for all *k* = 1, … , *r*, maximum number of iterations *M*_max_, regularization parameter precision δ_ϵ_, convergence precision δ_*x*_
2:	**initialization: J** ← **UV**^T^
3:	
4:	for *m* ← 1, … , *M*_max_ **do**
5:	**for** *k* ← 1, … , *r* **do**
6:	*a*′ ← *a*, **h**′ ← **h**, **U**′ ← **U**, **V**′ ← **V**
7:	$\text{J}\leftarrow \text{J}-{\text{U}}_{•,k}^{\prime }{\text{V}}_{•,k}^{\prime \text{T}}$ ⊳ remove block *k* from **J**
8:	λ_*L*_ ← max(|λ_max_(**∇**_**J**_*L*)|, |λ_min_(**∇**_**J**_*L*)|) evaluated with primed variables and **J**
9:	**do**
10:	ϵ_*k*_ ← λ_*L*_
11:	*a*′, **h**′, ${\text{U}}_{•,k}^{\prime }$, ${\text{V}}_{•,k}^{\prime }\leftarrow$ Solve the block *k* subproblem (Equation 13) using an
12:	interior-point method algorithm with inputs *a*′, **h**′, ${\text{U}}_{•,k}^{\prime }$, ${\text{V}}_{•,k}^{\prime }$, **J**,
13:	ϵ_*k*_, π_*k*_, (**s**_*t*_, *y*_*t*_):∀*t*
14:	λ_*L*_ ← max(|λ_max_(**∇**_**J**_*L*)|, |λ_min_(**∇**_**J**_*L*)|) ⊳ update eigenvalue threshold
15:	**while** ϵ_*k*_ ∉ [λ_*L*_, λ_*L*_ + δ_ϵ_]
16:	$\text{J}\leftarrow \text{J}+{\text{U}}_{•,k}^{\prime }{\text{V}}_{•,k}^{\prime \text{T}}$ ⊳ include *k*th block solution in **J**
17:	a ← *a*′, **h** ← **h**′, **U** ← **U**′, **V** ← **V**′
18:	**if** ${\|\text{x}-{\text{x}}^{\prime \|}}_{2}\leq \delta_{x}$ **and** {ϵ_*k*_:ϵ_*k*_ ∉ [λ_*L*_, λ_*L*_ + δ_ϵ_], ∀*k*} = ∅ **then**
19:	*(where* $\text{x}=\left[a,{\text{h}}^{\text{T}},{\text{Q}}_{•,1}^{\text{T}},\ldots \;,{\text{Q}}_{•,r}^{\text{T}}\right]$ *and* **x**′ *is the analogous vector for primed weights)*
20:	**break** ⊳ optimization has finished
21:	
22:	**outputs:** *a*, **h**, **J**

Our approach to the hyperparameter optimization in the locally optimal domain exploits the structure of the block coordinate descent subproblems (Equation 13) where the gradient and Hessian of the block *k* subproblem only depends explicitly on ϵ_*k*_. Holding the remaining **Q**_•,*j*_ (*j* ≠ *k*) fixed, the *k*th block is optimized while varying ϵ_*k*_ via a grid search on the domain ϵ_*k*_ ∈ [0, ϵ_max_] where ϵ_max_ is chosen to be large enough such that **Q**_•,*k*_ ≈ **0** when ϵ_*k*_ = ϵ_max_. We can estimate the generalization ability of the *i*th solution x^*(*i*)^ for a chosen value of the ϵ_*k*_ parameter, ϵ_*k*_*i*__ ∈ [0, ϵ_max_], by evaluating the negative log-likelihood $L_{\text{CV}}\left({\text{x}}^{*\left(i\right)}\right)$ where *y*_*t*_ and **s**_*t*_ are now samples drawn from the cross-validation set. If $L_{\text{CV}}\left({\text{x}}^{*\left(i\right)}\right)\leq L_{\text{CV}}\left({\text{x}}^{*\left(j\right)}\right)$ for all ϵ_*k*_*j*__ ∈ [0, ϵ_max_] of the block *k* subproblem (Equation 13), then x^*(*i*)^ is taken to be the most generalizeable estimate of the weights for the block *k* subproblem. The optimization completes when several full cycles through all *r* blocks of the block coordinate descent algorithm fail to provide a further decrease in *L*_CV_(**x**). For a pseudocode implementation, see Algorithm 2.

**Algorithm 2:** Low-rank MNE block coordinate descent algorithm (locally optimal domain)

1:	**inputs:** maximum rank *r*, maximum range for regularization parameters ϵ_max_, number of regularization parameter grid points *n*_grid_, training set indices *T*_train_⊆{1, … , *N*} and cross-validation set indices *T*_CV_⊂{1, … , *N*} where *T*_train_∩*T*_CV_ = ∅, paired data samples (**s**_*t*_, *y*_*t*_) for all *t* ∈ *T*_train_∪*T*_CV_, initial guess for weights *a*, **h**, **U**, and **V**, set π_*k*_ for all *k* = 1, … , *r*, maximum iterations *M*_max_, convergence precision δ_*p*_, maximum failures to find a better solution σ_max_
2:	**initialization: J** ← **UV**^T^, *L*_best_ ← *L*(*a*, **h**, **U**, **V**)|_*T*_CV__ (evaluated over data indices *t* ∈ *T*_CV_), regularization grid resolution δ_ϵ_ ← ϵ_max_/*n*_grid_, early completion switch σ ← 1
3:	
4:	**for** *m* ← 1, … , *M*_max_ **do**
5:	**for** *k* ← 1, … , *r* **do**
6:	*a*′ ← *a*, **h**′ ← **h**, ${\text{u}}^{\prime }\leftarrow {\text{U}}_{•,k}$, ${\text{v}}^{\prime }\leftarrow {\text{V}}_{•,k}$
7:	**J** ← **J** − **u**′ **v**′^T^ ⊳ remove block *k* from **J**
8:	**for** *n* ← 0, … , *n*_grid_ **do**
9:	ϵ_*k*_ ← *nδ*_ϵ_
10:	*a*′, **h**′, **u**′, **v**′ ← Solve the block *k* subproblem (Equation 13) using an
11:	interior-point method algorithm with inputs *a*′, **h**′, **u**′, **v**′, **J**,
12:	ϵ_*k*_, π_*k*_, (**s**_*t*_, *y*_*t*_):∀*t* ∈ *T*_train_
13:	$L\prime =L(a\prime ,\text{h}\prime ,\text{J}+\text{u}\prime \text{v}\prime^{\text{T}}){|}_{T_{\text{CV}}}$
14:	if $L^{\prime }<L_{\text{best}}-\delta_{p}$ **then**
15:	$L_{\text{best}}\leftarrow L^{\prime }$
16:	*a* ← *a*′, **h** ← **h**′, ${\text{U}}_{•,k}\leftarrow {\text{u}}^{\prime }$, ${\text{V}}_{•,k}\leftarrow {\text{v}}^{\prime }$
17:	σ ← 0
18:	**else if** $L^{\prime }\leq L\left(a,\text{h},\text{U},\text{V}\right){|}_{T_{\text{CV}}}$ **then**
19:	*a* ← *a*′, **h** ← **h**′, ${\text{U}}_{•,k}\leftarrow {\text{u}}^{\prime }$, ${\text{V}}_{•,k}\leftarrow {\text{v}}^{\prime }$ (or skip for monotonic convergence)
20:	$\text{J}\leftarrow \text{J}+{\text{U}}_{•,k}{\text{V}}_{•,k}^{\text{T}}$ ⊳ include block *k*'s solution in **J**
21:	**if** σ = σ_max_ **then**
22:	**break** ⊳ optimization has finished
23:	σ ← σ + 1
24:	
25:	**outputs:** *a*, **h**, **J**

We tested both of these algorithms and found the locally optimal domain to be most appropriate for recovering receptive field components. In the applications of low-rank MNE to model neurons and avian auditory neurons (for details about the data, see the methods section), we found the minimum amount of regularization necessary to reach the globally optimal domain was unreasonably large (ϵ_*k*_ ~ 1 or more). These large regularization parameters were found to severely attenuate the variance of the recovered components (i.e., the eigenvalues of **J**). For instance, the variance of the components of **J** reconstructed from the model neuron data was two orders of magnitude lower than the ground truth. This attenuation was accompanied by substantial distortion of the components. Similarly, solutions that were found in the locally optimal domain had much better generalization ability across both the model neurons and the avian neurons as measured by evaluating the negative log-likelihood on the cross-validation sets.

The global optimization procedure outlined in Algorithm 1 runs very quickly, usually finding a solution within 1–4 hours for problems of size *D* = 400 to 1, 200 and *r* = 1 to *r* = 20 (see Section 4.8 for hardware/software details). The local optimization procedure in Algorithm 2, on the other hand, can range from on the order of less than an hour to a day for problems of size *D* = 400 to 1, 200 and *r* = 1 to *r* = 20. It should be said, however, that the goal of these algorithms are to find good solutions to Equation (8) but we made little attempt to optimize these algorithms for speed. There are two primary bottlenecks in the optimization: (i) the choice of subproblem solver and (ii) the choice of hyperparameters to use in the optimization. Since the optimization procedure is highly customizeable, the timing of these bottlenecks will be highly variable on the choices made by the investigator. For instance, solving the block subproblem may be sped-up by using L-BFGS instead of the exact Hessian on the larger *D* problems. Furthermore, there are other approaches that may be taken in place of Algorithm 2 to choose hyperparameters. In particular, one can replace the blockwise grid search with a random search for the hyperparameter settings (Bergstra and Bengio, 2012) or use Bayesian optimization (Brochu et al., 2010; Snoek et al., 2012). We performed some preliminary analysis using Bayesian optimization and found it to be a competitive alternative to Algorithm 2 that may speed up the optimization for large *D*.

#### 2.1.4. Rank optimization

Depending on the application, there are a several possible ways to choose the rank of **J** in the low-rank MNE model. For instance, one may intend to find the optimal rank of **J**, *r*_opt_, defined as the rank of **J** of the model that has the best generalization performance to novel data. In this instance, an unregularized model would be fit by trying different signs, π_*k*_, for the constraints and maximum rank *r* and then choose the *r*_opt_ model as that which makes the best predictions on novel data. For a nuclear-norm regularized model, the fit is more flexible since *r* can instead be treated as an upper bound on the rank of **J** while the regularization can be used to lower the rank, if necessary. In this case, *r*_opt_ can instead be determined by finding some model of maximum rank *r* where **J** is rank-deficient with respect to at least one π_*k*_ = 1 and π_*k*_ = −1 constraint as determined by the number of negative and positive eigenvalues of **J**. The justification for this approach is that if the regularization procedure leads to **U**_•,*r*_opt_+1_ = **V**_•,*r*_opt_+1_ = **0** for trials with both signs of π_*k*_ = ±1, the value of the cost function (*f*) is left unchanged from the *r*_opt_ model. Adding additional columns in **U** and **V** beyond *r*_opt_ + 1 would be equivalent to optimizing the *r*_opt_ + 1 model. Procedurally, one can guess *r* that is ostensibly an upper bound on *r*_opt_ and if there is at least one vector **Q**_•,*i*_ = **0** for π_*i*_ = −1 and at least one vector **Q**_•,*j*_ = **0** for π_*j*_ = 1 that is zero in **Q**, then *r*_opt_ = rank(**Q**). This is equivalent to splitting **J** into a sum of a positive semidefinite and negative semidefinite matrix, **J** = **J**_psd_ + **J**_nsd_, where the optimal rank would be rank(**J**) when both **J**_psd_ and **J**_nsd_ are composed of rank-deficient bilinear factorization matrices (i.e., rank(**Q**_psd_) < *r*_psd_ and rank(**Q**_nsd_) < *r*_nsd_). If this condition is not met, however, the maximum rank, *r*, must be increased and the optimization must continue with extra columns appended to **Q** and each π_*k*_ set appropriately until this condition is met. Since we are looking for the optimal rank in our applications, we use this procedure for determining the rank. We initialize the π_*k*_ parameters such that **J**_psd_ and **J**_nsd_ have equal maximum rank.

If instead one intends to find a compressed representation of **J** where *r* < *r*_opt_, the only remaining unset parameters are π_*k*_. This can be done by solving the problems with different choices of π_*k*_ and keeping the model that fits the best either to the training or cross-validation sets. Instead of solving models that enumerate all possible choices of π_*k*_ for all *k* = 1…*r*, one can instead take a shortcut by solving lower-rank models of rank *r*_*n*_, incrementing the rank of the model (e.g., *r*_*n* + 1_ = *r*_*n*_ + 1), enumerating solutions with π_*k*_ fixed for all *k* ≤ *r*_*n*_, and repeating until reaching a rank *r* model. Alternatively, one can also use other principled means for choosing π_*k*_ including the eigenvalues of **J** from the full-rank MNE model or from an unconstrainted low-rank MNE model. One may also attempt to do away with the π_*k*_ parameters entirely by using one of the alternative constraint formulations (Section 5.1 in the Appendix).

### 2.2. Testing the algorithm on model neurons

**Figure 1:**
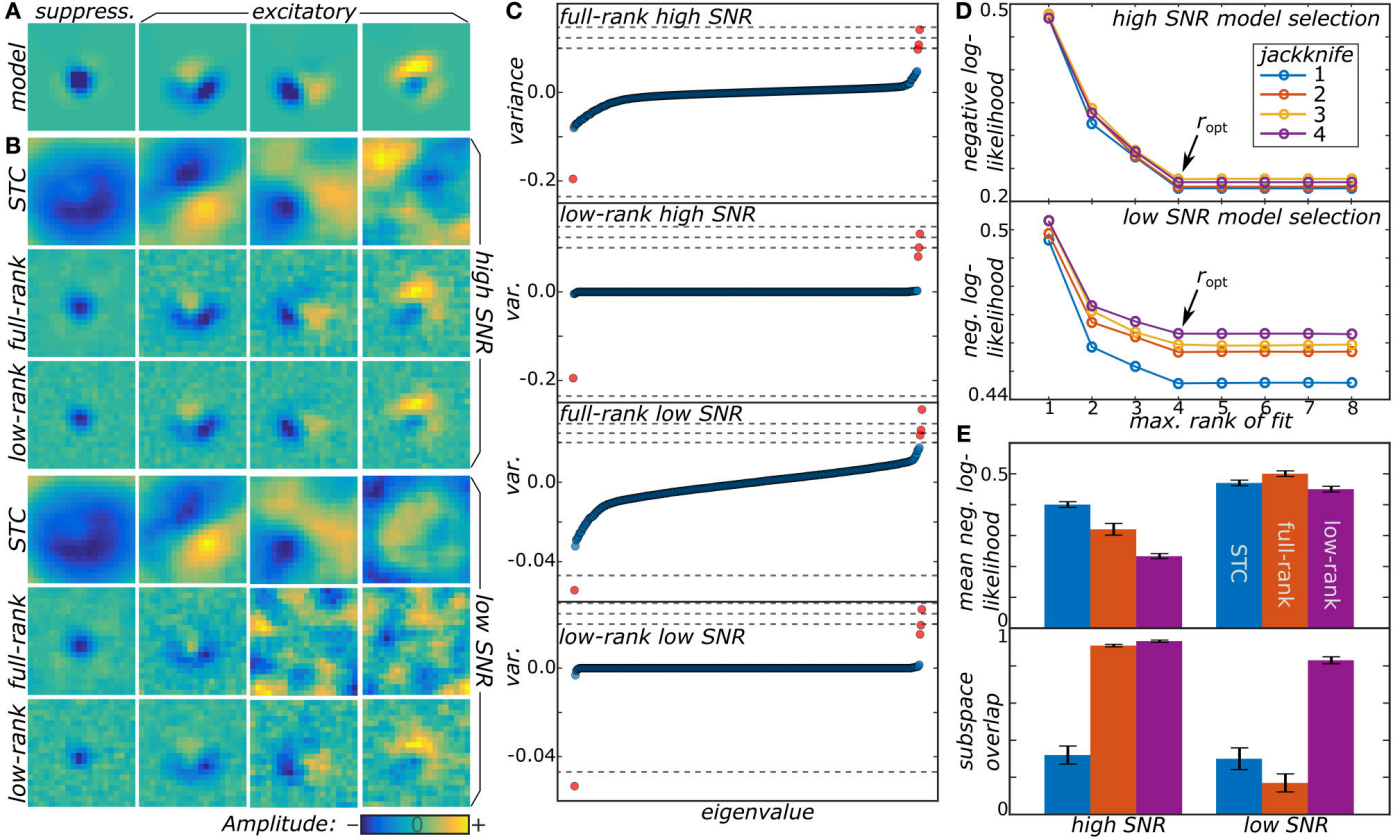
**(A)** Two model neurons were generated from a synthetic receptive field, one with high SNR and another with low SNR. **(B)** Low-rank MNE, full-rank MNE, and STC models were optimized for both of the neurons and the mean top four largest magnitude components of **J** were plotted. **(C)** These mean components correspond to the largest variance eigenvalues in the eigenvalue spectra. The dashed lines in the eigenvalue spectra correspond to the eigenvalues of the ground truth, **J**_GT_. **(D)** Low-rank models with maximum rank ranging over *r* = 1…8 were trained on four different jackknives of the data set where for each jackknife the data set was split into a training and cross-validation set. The predictive power of each jackknife's trained models evaluated on its cross-validation set is shown to saturate when *r* ≥ *r*_opt_ = 4. **(E)** Quantitative comparisons of the receptive field reconstructions from the three methods is compared based on each model's predictive power on the test sets (top) and the subspace overlap onto the ground truth (bottom).

We now illustrate the proposed method by analyzing responses of model neurons. Details about the model data may be found in Section 4.4 in methods. First, we tested the method on two model neurons with different signal-to-noise ratios (SNR) (cf. Figure 1). Both the low-rank and full-rank approaches yielded good reconstructions in the high SNR regime, finding all of the four relevant components of the model. The subspace overlap (Equation 22 in methods) between the set of model and reconstructed dimensions was 0.933 ± 0.007 and 0.909 ± 0.008 for the low and full-rank approaches, respectively. The STC method that is standard for noise-like stimuli (Schwartz et al., 2006) performs worse here, because it is not designed to work with stimuli drawn from correlated distributions, with subspace overlap of 0.32 ± 0.05. We note that although the low-rank and full-rank approaches recover the component subspace with reasonable accuracy, the low-rank models produce much more accurate predictions on the test sets (0.233 ± 0.009 vs. 0.32 ± 0.02 for the negative log-likelihood of low-rank and full-rank models, respectively). The main advantage of the low-rank approach becomes apparent in the ultra-low SNR regime. Here, the full-rank model failed to recover all of the relevant components finding only two out of four with a subspace overlap of 0.17 ± 0.05. In contrast, the low-rank model correctly determined the number of relevant components with a subspace overlap of 0.83 ± 0.02. This is much better than the STC method where the subspace overlap was 0.30 ± 0.06.

From a qualitative point of view, the low-rank model performs better than the full-rank model because the matrix **J** is less corrupted by noise present in the data. This can be seen by looking at the eigenvalue spectra of **J** in Figure 1C where the full-rank models recover a nearly full-rank **J** matrix dominated by fictitious components. The large number of fictitious components contribute substantially to the variance of **J** leading to an overall decrease in the predictive power of the model. The significance of these fictitious components becomes even more substantial in the low SNR regime where their eigenvalues nearly engulf the eigenvalues of the relevant components. By contrast, the low-rank models exhibit a sparse eigenvalue spectrum of rank consistent with the ground truth in Figure 1A.

We also found fits of the low-rank model to be resilient even when the rank of **U** and **V** was larger than the ground truth. For example, in Figure 1D, we show the results for fitting low-rank models with rank *r* = 1, … , 8 (using signs of the eigenvalues of the mean **J** matrix from the full-rank models to initialize the π_*k*_ values). Here, the negative log-likelihood evaluated on the cross-validation set saturates as *r* becomes greater than or equal to the ground truth value of 4. Above *r* = 4, the regularization procedure eliminates the fictitious dimensions that infected the full-rank models leading to a rank-deficient solution equivalent to the *r* = 4 model. On the other hand, the models with *r* < 4 have a higher negative log-likelihood because, by design, they cannot recover all four components and represent a low-rank compression of **J**.

### 2.3. Application to avian auditory data

**Figure 2:**
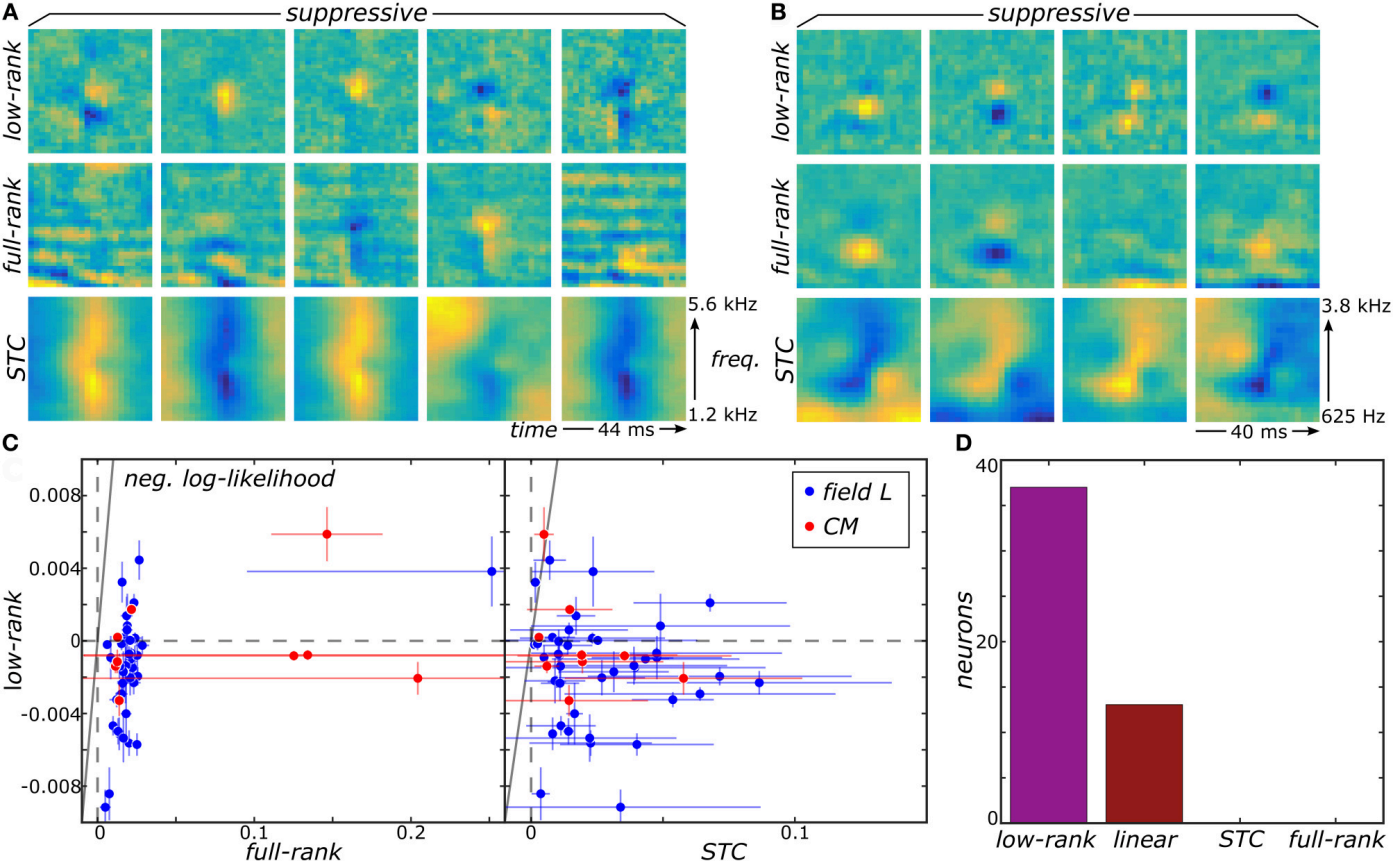
Low-rank MNE, full-rank MNE, and STC models were optimized on a dataset of 50 avian auditory forebrain neurons. Logical OR and logical AND FB models were fit using linear combinations of the subspace components of each method. Logical AND FB models from two example neurons are shown **(A,B)**. The quality of each model is measured using the difference between the mean negative log-likelihood of the model and the linear MNE model evaluated on the test sets and plots summarize predictive ability across the population of neurons **(C)**. A bar plot quantifies the number of neurons in the population best fit by each model **(D)**. Note low-rank and linear MNE models outperform all STC and full-rank MNE models across the population.

We now show that the proposed low-rank MNE method offers substantial improvement in our ability to resolve multiple relevant components of sensory neurons' receptive fields by applying it to recordings from the avian auditory forebrain (Gill et al., 2006; Amin et al., 2010). For details about these recordings and data processing, see Section 4.5 in methods. First, the low-rank method produces much sharper components that are more localized in both frequency and time compared to components of the full-rank estimation (Figures 2A,B). The improvement over the STC components is even more dramatic (Figures 2A,B). This difference becomes more pronounced for components that account for lower variance in the neural response. For such components, the low-rank method can resolve localized regions of sensitivity under the broad bands that dominate in the full-rank method, e.g., for components in columns 2–4 in Figure 2A. Quantitatively, reconstructions of neural responses obtained with low-rank models yield universally higher predictive power on novel data subsets compared to the full-rank and STC models (Figure 2C). Importantly, the full-rank models did not yield better predictions over the linear one-component MNE models (**J** = **0**) for all neurons. Therefore, the additional variables in the full-rank model do not yield any statistically significant components because the full-rank model does not improve predictions on the test sets relative to the linear model. This is despite the leading components of the full-rank reconstructions bearing apparent similarity to the top components of the low-rank reconstruction. STC models made worse predictions than the linear MNE models across all neurons as well. By comparison, the low-rank optimization yielded better predictions on the test sets compared to the linear model for 37 of the 50 neurons (Figure 2D).

**Figure 3:**
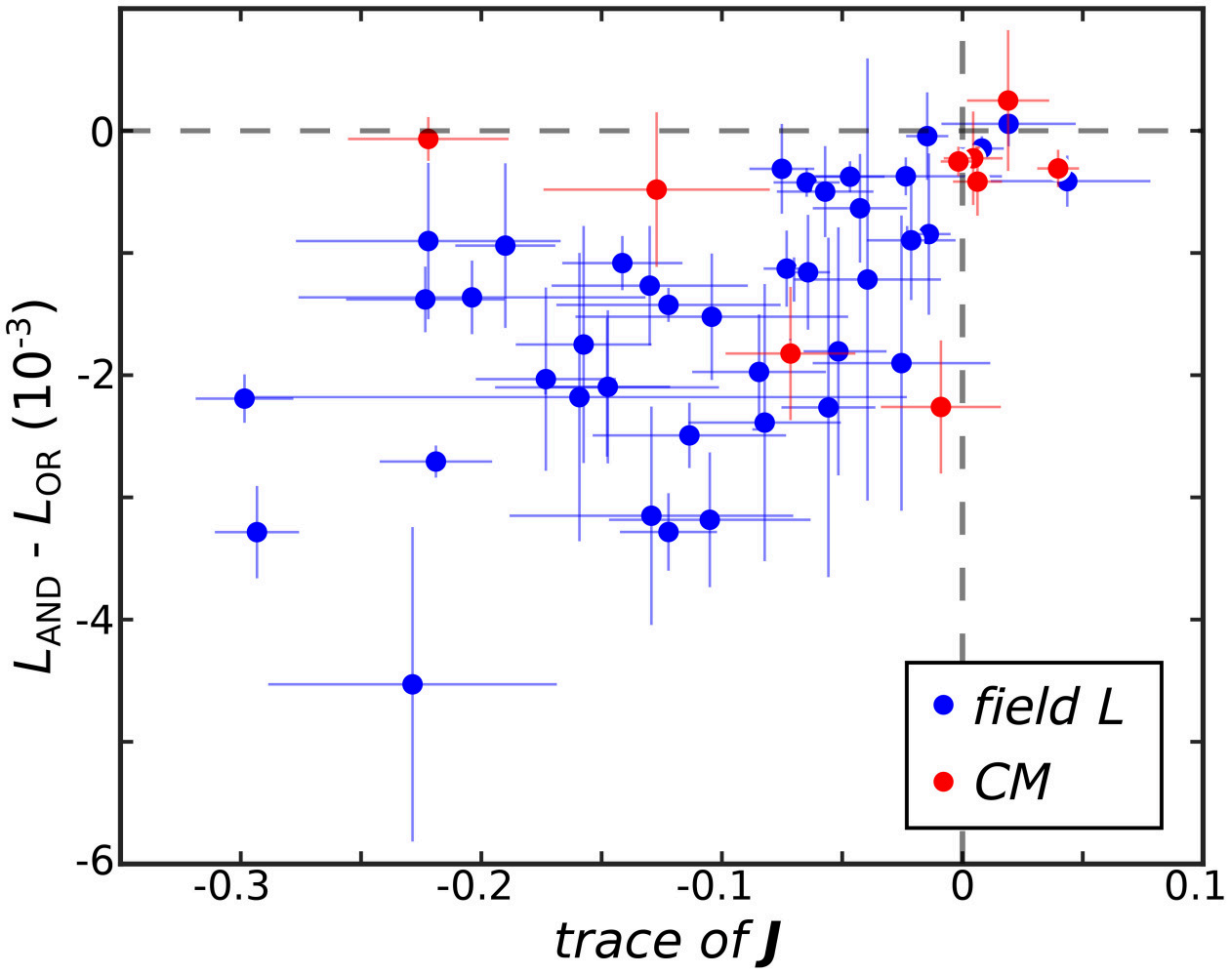
The difference between the negative log-likelihood of the best logical AND (*L*_AND_) and logical OR (*L*_OR_) models averaged across test sets is plotted against Tr(**J**) where here **J** is averaged across jackknives. The horizontal dashed line demarcates neurons best fit by logical OR models on top from neurons best fit by logical AND models below. The vertical dashed line separates neurons where the eigenvalue spectra of mean **J** are dominated by negative variance on the left and positive variance on the right.

The ultimate utility of methods for receptive field reconstruction is to produce models that can inform our understanding of the transformations performed by high-level sensory neurons. Toward that goal, one can subject the components obtained from low-rank reconstructions to a functional basis (FB) transformation (Kaardal et al., 2013). This transformation aims to account for the observed neural responses in terms of logical operations, such as logical AND/OR, on the set of input components. By studying whether populations of neurons are best fit by logical AND/OR functions, we can learn whether populations of neurons in certain regions of the brain compute primarily conjunctive or integrative functions of their inputs. A conjunctive neuron would be selective toward coincidences of multiple relevant inputs and corresponds to a logical AND function. An integrative neuron is responsive toward any relevant input and corresponds to a logical OR function. Here we find that FB models based on logical AND combinations overwhelmingly outperformed models based on logical OR across the population where 40 of the 41 field L neurons and 8 of the 9 CM neurons were best fit by logical AND models (Figure 3). To gain intuitive understanding for these results, we note that a logical AND operation is equivalent to a logical OR followed by negation. These results therefore suggest that logical AND better represents cases where invariances are built into suppressive receptive field components. This is because logical OR combinations often work well to approximate invariance in neural responses that occur if any relevant stimulus features are present, corresponding to the logical OR operation (i.e., if **v**_*k*_ · **s**_*t*_ for *any k* is greater than some threshold, where **v**_*k*_ is a receptive field component, the neuron spikes in response to sample *t*). When these components are all suppressive, this implies that the neural response occurs if none of the relevant features are present in the stimulus (i.e., if **v**_*k*_ · **s**_*t*_ for *any k* is greater than some threshold, the neuron is silent at sample *t*). This would correspond to the logical AND model. Supporting these arguments, we found neurons with stronger suppressive components were better described by logical AND models over logical OR models (Figure 3) with a t-test p-value of 0.1%.

## 3. Discussion

By using low-rank MNE models that are resistant to both overfitting and the biases of naturalistic stimuli, we have estimated multiple components relevant to responses of neurons from the avian auditory forebrain with much greater accuracy than by prior methods. Interestingly, we found that receptive fields of neurons from field L and CM, relatively high-level regions of the avian auditory forebrain, were composed of few components (*r* ≤ 20). This number is small enough for the resultant models to provide interpretable representations of the underlying receptive fields.

We demonstrated that the low-rank MNE models produced better predictive models than full-rank MNE, STC, and linear MNE models and did so with a fewer components than the full-rank MNE models across the population of neurons. There are several reasons why this improvement is observed. As mentioned before, MNE models in principle produce better reconstructions of the relevant components than STC for stimuli drawn from distributions other than Gaussian white noise. This was demonstrated in practice where we saw the STC models performing worse than the low-rank and linear MNE models on both model neurons and recordings from auditory neurons subject to correlated stimuli. With regard to the full-rank MNE models, the low-rank MNE models have three major advantages: (i) the number of components necessary to optimize is explicitly reduced, (ii) the optimization procedure uses nuclear-norm regularization to eliminate fictitious components, and (iii) significant components are recovered via a nonlinear matrix factorization. The first reason is simply a matter of reducing overfitting since fewer weights were estimated in the low-rank MNE models than the full-rank MNE models. To the second point, both the full-rank and low-rank MNE methods used a form of regularization as an attempt to reduce this overfitting but the early exiting procedure used by the full-rank MNE method (Fitzgerald et al., 2011a) did not impose defined structure on **J** while the low-rank MNE regularization procedure directly eliminated components from **J**. Lastly, while it may be approximately true in many cases, one cannot generally assume that the size of the contribution of each component of **J** toward the predictive power of an MNE model will correspond to the variance of the component. In fact, we observed from randomly generated low-rank MNE problems with suboptimal local minima that an optimal component corresponding to highest variance was not necessarily the component that led to the best fit. This seems likely to be an important consideration for other nonlinear matrix factorization methods as well. In contrast, linear matrix factorizations like in the STC method where each component is a local minimum of the matrix factorization has a direct correspondence between the variance of the component and quality of the low-rank fit.

Ultimately, since the optimization of full-rank MNE models is convex, the weights that minimize the negative log-likelihood represent the ground truth as captured by the training data. Thus, if the full-rank MNE model's representation of **J** is high or even full-rank, the global solution of the low-rank MNE optimization problem in the absence of regularization would also be high or full-rank. In such cases, including our applications, it is unsurprising that the locally optimal domain would produce solutions that generalize significantly better than the globally optimal domain for low-rank representations of **J**. This is because the solutions in the globally optimal domain must have large enough regularization parameters to eliminate all possible higher-rank solutions; a requirement that is relaxed in the locally optimal domain.

Overall, the efficiency of the low-rank optimization for the extraction of multiple input components makes it possible to begin to resolve a long-standing puzzle of how high-level auditory responses can combine selectivity to sharp transients with integration over broad temporal components. We find that it is possible to reconstruct many more components more accurately than was possible before. The results highlight an interesting potential difference between high-level visual and auditory responses. In vision, current studies (Serre et al., 2007; Kaardal et al., 2013) show that logical OR models are better at describing the neural computations while this initial analysis suggests that logical AND operations are better at explaining responses in the avian auditory forebrain. It is worth noting that both logical OR and logical AND models could indicate the presence of invariance to certain stimulus transformations. The difference is that logical OR models would capture invariance constructed by max pooling responses among excitatory dimensions whereas logical AND would capture invariance constructed by max pooling among suppressive dimensions. Here, pooling is used as an approximation to logical OR; when applied to suppressive dimensions it converts to a logical AND operation because of negation (that is, in the logical AND model the response is observed if the stimulus has no features that simultaneously strongly project onto any of the receptive field components). Thus, one arrives at a potential important difference between visual and auditory processing. In the visual system (Serre et al., 2007), invariance is achieved by max pooling across primary excitatory dimensions whereas in the auditory system invariance is achieved by suppression.

## 4. Methods

### 4.1. The first-order and full-rank MNE methods

The full-rank MNE method (Fitzgerald et al., 2011a) solves the convex, nonlinear program:

(15) $$\begin{array}{l} \underset{a,\text{h},\text{J}}{\min}L(a,\text{h},\text{J}) \end{array}$$

where *L* is the negative log-likelihood from before (Equation 2) but with **J** directly optimized and without explicit regularization. Since the full-rank MNE problem is convex, we find a global optimum of *L* via conjugate gradient descent. As a mild form of regularization, early stopping is used where the performance of the model after each conjugate gradient descent step is measured on a cross-validation set and the algorithm returns the weights *a*, **h**, and **J** with minimal negative log-likelihood evaluated on the cross-validation set. The early stopping criterion is to halt optimization after 40 consecutive iterations of the conjugate gradient descent algorithm fail to decrease the negative log-likelihood evaluated on the cross-validation set. The quadratic weights that form a subspace relevant to the neural response are extracted via eigendecomposition in the same way as the low-rank method (Equation 11).

First-order MNE models solve (Equation 15) with **J** fixed to zero. The receptive field is approximated entirely by the linear weights, **h**. Since the first-order MNE method is also convex, it is fit using conjugate gradient descent with early stopping using the exact same procedure as the full-rank MNE method.

### 4.2. Spike-triggered average (STA) and spike-triggered covariance (STC) methods

The STA and STC methods are standard methods for analyzing receptive fields of neurons stimulated by Gaussian white noise stimuli (Schwartz et al., 2006). To calculate the STA, the difference between a spike-weighted average of zero-centered stimuli is computed:

(16) $$\begin{array}{l} {\text{h}}_{\text{STA}}=\frac{1}{N_{\text{spk}}}\sum_{t=1}^{N}y_{t}{\text{s}}_{t}, \end{array}$$

where **h**_STA_ is a single component estimate of the receptive field. Note that the STA can only compute one component. STC, on the other hand, can estimate multiple components of the receptive field. For STC, the difference of two covariance matrices is calculated:

(17) $$\begin{array}{l} \text{C}=\frac{1}{N_{\text{spk}}}\sum_{t=1}^{N}y_{t}{\text{s}}_{t}{\text{s}}_{t}^{\text{T}}-\frac{1}{N}\sum_{t=1}^{N}{\text{s}}_{t}{\text{s}}_{t}^{\text{T}}, \end{array}$$

one the spike-weighted mean stimulus covariance and the other the mean stimulus covariance. As with the STA, the stimuli are zero-centered. The matrix **C** is diagonalized:

(18) $$\begin{array}{l} \text{C}=\Omega \Sigma \Omega^{\text{T}} \end{array}$$

and the relevant components are spanned by the eigenvectors corresponding to the largest variance eigenvalues. Determining where to cut-off the eigenvalue spectrum is done by randomly shuffling the responses in the training set to break correlations between the stimuli and responses (Bialek and de Ruyter van Steveninck, 2005; Rust et al., 2005; Schwartz et al., 2006; Oliver and Gallant, 2010) and then generating randomized STC matrices, **C**_rand_, from Equation (17). All eigenvalues of **C** with larger variance than the largest variance eigenvalue of **C**_rand_ are considered significant and form the estimate of the relevant subspace.

Since STC is not equipped with a nonlinearity, we optimize a full-rank MNE model with all **s**_*t*_ projected into the relevant components from above and use the resulting weights to estimate the predictive power of the model on the test set. If **Ω**_*r*_ is the rank *r* STC basis, then the stimulus space is transformed into the reduced stimulus space, ${\text{s}}_{t}^{\left(\text{red}\right)}=\Omega_{r}^{\text{T}}{\text{s}}_{t}$, and then the full-rank MNE problem (Equation 15) is minimized on the training set projected into this reduced stimulus space.

To contend with the strong correlations present in the data sets, we repeated the above STC analysis with stimulus correlations removed through data whitening. Data whitening removes the mean correlations between elements of the stimulus space such that the mean covariance of the stimulus samples is the identity matrix. This can be a beneficial pre-processing step for STC models since STC models are biased when the stimulus space is not Gaussian white noise distributed. There are standard ways of whitening data known as principal component analysis (PCA) and zero-phase (ZCA) whitening (Bell and Sejnowski, 1997), both of which we implemented. In both cases, the first step is to take the singular-value decomposition of the mean centered (mean stimulus vector subtracted) stimulus covariance matrix:

(19) $$\begin{array}{l} \frac{1}{N}\sum_{t=1}^{N}\left(s_{t}-\langle s_{t}\rangle \right){\left(s_{t}-\langle s_{t}\rangle \right)}^{\text{T}}=LEL^{\text{T}}. \end{array}$$

Then, for the PCA whitening transform the stimuli are transformed as ${\text{s}}_{t}^{\left(\text{PCA}\right)}={\text{E}}^{-\frac{1}{2}}{\text{L}}^{\text{T}}{\text{s}}_{t}$ while the ZCA whitening transforms the stimuli as ${\text{s}}_{t}^{\left(\text{ZCA}\right)}=\text{L}{\text{E}}^{-\frac{1}{2}}{\text{L}}^{\text{T}}{\text{s}}_{t}$. From here, the procedure is the same as before with the whitened stimuli substituted for **s**_*t*_. We observed, however, that these decorrelation methods performed poorly, producing receptive fields that lacked discernible structure and with worse predictive power compared to standard STC.

### 4.3. Functional basis method

The FB method (Kaardal et al., 2013) was used to recover biologically interpretable characterizations of the receptive field. This is done by modeling the nonlinearity as logical circuit elements where a linear combination of the inputs determine the probability of a spike. The two most basic descriptions are logical AND:

(20) $$\begin{array}{l} P_{\text{AND}}(y=1|{\text{s}}_{t})=\prod_{k}\frac{1}{1+e^{-b_{k}-\zeta_{1}{\text{c}}_{k}^{\text{T}}{\text{s}}_{t}-\zeta_{2}{\left({\text{c}}_{k}^{\text{T}}{\text{s}}_{t}\right)}^{2}}}, \end{array}$$

and logical OR:

(21) $$\begin{array}{l} P_{\text{OR}}(y=1|{\text{s}}_{t})=1-\prod_{k}\left[1-\frac{1}{1+e^{-b_{k}-\zeta_{1}{\text{c}}_{k}^{\text{T}}{\text{s}}_{t}-\zeta_{2}{\left({\text{c}}_{k}^{\text{T}}{\text{s}}_{t}\right)}^{2}}}\right], \end{array}$$

where *b*_*k*_ are thresholds, ζ_1_ is a linear weighting, ζ_2_ is a quadratic weighting, and **c**_*k*_ are FB vectors that are formed by taking linear combinations of the relevant components from **Ω** (Equation 11). The functional basis is fit by minimizing the negative log-likelihood using an L-BFGS algorithm. However, since it is a nonconvex optimization and therefore not guaranteed to converge to a global minimum, the optimization is repeated with multiple random initializations until 50 consecutive optimizations fail to produce a model that better fits the training set data. The FB model that best fits the training data is returned.

In prior applications, the FB method has produced basis vector spaces that are equal to *r*_opt_. However, this need not be the case and the FB method may yield a basis with more or less dimensions than the underlying subspace. The optimal basis size can be determined by varying the number of components until the negative log-likelihood evaluated on the cross-validation set saturates to desired accuracy. The minimal number of components necessary to reach saturation is the desired number of basis vectors.

### 4.4. Synthetic data

The low-rank MNE method was tested on synthetic data generated from model receptive fields: (i) a neuron with a high SNR and (ii) a neuron with a low SNR. In this case, high and low SNR correspond to the relative decisiveness of each model neuron's response to a stimulus. For instance, a high SNR model neuron is more likely to have a nonlinearity where *P*(*y* = 1|**s**) is close to 0 or 1 compared to a low SNR model neuron which is more likely to take on intermediate probabilities. Using Equation (1), high and low SNR model neurons can be generated from a given set of weights by adjusting the gain of *z*. Concretely, the model neurons were generated by taking a sum of the weighted outer-products of the orthonormal vectors stored in the 400 × 4 (*D* × *r*_opt_) matrix **F** (Figure 1A) yielding the ground truth matrix, ${\text{J}}_{\text{GT}}=\text{F}\text{W}{\text{F}}^{\text{T}}$. The linear weights, **h**_GT_, were set to zero while the threshold, *a*_GT_, and the 4 × 4 diagonal weight matrix, **W**, were independently rescaled to produce a mean firing rate 〈*y*〉≈0.2 by averaging *P*(*y* = 1|**s**_*t*_) over *t* where **s**_*t*_ is the *t*th 20 × 20 pixel stimulus sample drawn from a correlated Gaussian distribution unrolled into a *D* = 400 vector. The diagonal elements of the rescaled weight matrix, **W**, appear as the dashed lines in Figure 1C and correspond to the eigenvalues of **J**_GT_. Note that the eigenvalues of **J**_GT_ have larger variance for the high SNR model neuron compared to the low SNR model neuron which corresponds to the high SNR model having a higher gain.

**Figure 4:**
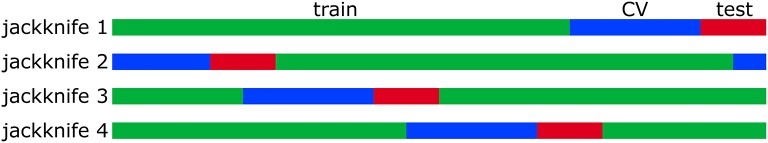
The data samples for each neuron are divided into 70% training (green), 20% cross-validation (blue), and 10% test (red) sets. The samples that appear in each set are varied between 4 jackknives.

The correlated Gaussian stimuli were generated by first drawing sample vectors, ${\hat{\text{s}}}_{t}$, from a normal distribution with zero mean and unit variance. A correlated Gaussian stimulus vector is then obtained via ${\text{s}}_{t}={\text{C}}_{\text{cov}}^{\frac{1}{2}}{\hat{\text{s}}}_{t}$ where **C**_cov_ is a covariance matrix constructed from natural images. Explicitly, ${\text{C}}_{\text{cov}}=\frac{1}{n_{\text{samp}}}\overset{n_{\text{samp}}}{\underset{t=1}{\sum }}\kappa_{t}\kappa_{t}^{\text{T}}$ where **κ**_*t*_ is the *t*th sample of a total of *n*_samp_ samples drawn from a set of 20 × 20 images unrolled into vectors. The responses were binarized into spikes by generating a list of uniformly distributed random numbers, **ξ**_*t*_ ∈ [0, 1]. If ξ_*t*_ < *P*(*y* = 1|**s**_*t*_), then *y*_*t*_ = 1; otherwise, *y*_*t*_ = 0. The total number of spikes was 11,031 for the high SNR model and 10,434 for the low SNR model. The same 48,510 stimulus samples were used as stimulus input to the model neurons. All models (first-order MNE, low-rank MNE, full-rank MNE, and STC models) were trained, cross-validated, and tested on 70%/20%/10% nonintersecting subsets of the data samples, respectively. In these proportions, the results were validated via jackknife analysis where four training, cross-validation, and test sets were defined by circularly shifting the sample indices in each set upward by 25% intervals of the total number of samples in the set (*t* ← *t* + *N*/4; see Figure 4).

### 4.5. Avian data

Data from the CRCNS database provided by the Theunissen laboratory was composed of *in vivo* electrophysiological recordings from anesthetized adult male zebra finches subjected to auditory stimuli (Gill et al., 2006; Amin et al., 2010). The recordings captured single-neuron action potentials from the auditory mid-brain, specifically 143 neurons from the mesencephalicus lateral dorsalis (MLd), 59 neurons from the ovoidalis (Ov), 37 neurons from the caudal mesopallium (CM), and 189 neurons from field L (L); the latter two of which are the focus of this paper. The temporal sampling resolution of the response was 1 ms. Two types of auditory recordings were used to stimulate action potentials: (1) 2 s samples of conspecific birdsong from 20 male zebra finches and (2) 10 synthetic recordings composed of sums of spectro-temporal ripples. Both stimulus types were bandpass filtered between 250 Hz and 8 kHz. These stimuli were presented to the zebra finches through speakers in a sound-attenuation chamber and each stimulus was repeated up to 10 times.

We processed the stimuli using MATLAB's spectrogram function on each sound clip and adjusted the frequency resolution of the spectrograms to find a reasonably well balanced compromise between the frequency and temporal resolution. Each Hamming window of the spectrogram had a 50% overlap with its neighbors. We found that a 250 Hz frequency resolution, which is coupled with a 2 ms temporal resolution, demonstrated a reasonable spectro-temporal resolution in the linear weights (**h**) of first-order MNE models where structure of the receptive field could be resolved and there were plenty of stimulus/response pairs for model fitting. The spike times, each corresponding to one spike, were accumulated in 2 ms bins across all trials of an auditory recording. Any 2 ms period without any spikes was set to zero. Since the number of spikes in a bin could be greater than one, the spike count was divided by the maximum number of spikes across temporal bins *y*_max_ = max(*y*_1_, … , *y*_*N*_), (*y*_*t*_ ← *y*_*t*_/*y*_max_). This ensured that the binned response was within the required range *y*_*t*_ ∈ [0, 1] and effectively corresponds to reducing the bin width by *y*_max_. The stimulus samples were assigned by extracting 40–60 ms windows from the spectrogram preceding the neural response at *y*_*t*_, excluding frequency bins well above and below the receptive field structures observed in **h**, and unrolling each spectro-temporal window into a stimulus feature vector, **s**_*t*_. The response/stimulus pairs were then randomly shuffled to ensure that the training sets each provided a wide sampling of the response/stimulus distribution.

We selected 41 of the 189 field L neurons and 9 of the 37 CM neurons for analysis. These neurons were chosen based on whether a spectro-temporal window of the stimuli could be identified that produced an estimate of a single-component receptive field with an observable structure. For each neuron, the STA (Schwartz et al., 2006) and first-order MNE methods were used to extract this component and the spectro-temporal window for each neuron was manually adjusted such that the estimated receptive field amplitude was confined to the spectro-temporal window. We did not use STC or second-order MNE methods for this pre-processing step because the computation of these second-order methods are relatively slow compared to the aforementioned first-order methods and the first-order methods appear to produce a single component that is a weighted average of the second-order components. Since the stimulus samples were spectro-temporally correlated, the STA suffered from bias leading to single component receptive field estimates covering excessively large spectral and temporal ranges when compared to the smaller spectro-temporal extent of the more appropriate MNE models. Furthermore, the STA was prone to yielding structure even when the temporal window was set far (e.g., >100 ms) from the spike-onset time where the MNE methods found no observable structure. By contrast, we found the first-order MNE method to be more reliable and less misleading, so first-order MNE was ultimately chosen to determine the spectro-temporal windowing of the neurons. The chosen neurons were then those that exhibited structure in the first-order MNE receptive field estimate determined by visual inspection. This procedure if anything biases the results toward greater performance of linear models. Despite this potential bias, we found that low-rank models outperformed the models based on one component. It is possible the relative improvement would be greater for other neurons not considered here.

The number of stimulus samples ranges from 9,800 to 58,169 with a median sample size of 42,474 and the spike counts are between 276 and 29,121 with a median count of 6,120. First-order MNE, low-rank MNE, full-rank MNE, STC, and FB models were trained, cross-validated, and tested on 70%/20%/10% of the data, respectively, over four jackknives incremented in the same way as was done with regard to the model neuron data (i.e., Figure 4).

### 4.6. Data analysis

Since we knew already that *r*_opt_ = 4 for the model neurons, we fit all *r* = 1, … , 8 low-rank MNE models demonstrating saturation of cross-validation performance at a *r* = *r*_opt_ = 4 model. For the avian data, *r*_opt_ was not known *a priori* so we instead fit low-rank MNE models with a maximum rank of *r* = 20 which satisfied the conditions set by the rank optimization section (above) where less than 10 of each positive and negative eigenvalues exceeded a magnitude greater than 1 · 10^−4^ on the majority of all jackknives for each neuron. A summary of the specific parameters used in Algorithm 2 to solve the low-rank MNE problems may be found in Table 1. We fit low-rank MNE models using both the stricter monotonic convergence approach and the looser nonmonotonic convergence approach in Algorithm 2 and found the difference in predictive power on the test sets between the two models to be insignificant. However, the nonmonotonic convergence approach had a tendency to produce sparser eigenvalue spectra of **J** so we opted to present the results from this version of the algorithm instead.

**Table 1:** Summary of parameter values used in our application of Algorithm 2.

**Parameter**	**Value**	**Definition**
*r*	Varies	Maximum rank of **J**
π_1_, … , π_*r*_	Varies	Constraint signs
ϵ_max_	0.5	Maximum value of the regularization parameters
*n*_grid_	501	Number of different values the regularization parameter can assume forming a uniform grid from 0 to ϵ_max_
*T*_train_	70% of samples	Indices of data samples that form the training set
*T*_CV_	20% of samples	Indices of data samples that form the cross-validation set
*M*_max_	20	Maximum number of iterations of the block coordinate descent algorithm
δ_p_	0 (machine precision)	Convergence precision
σ_max_	3	Number of allowed failures to improve cross-validation performance

*Since the convergence precision, δ_p_, is set to zero, the algorithm converges when the negative log-likelihood evaluated on the cross-validation set does not decrease above machine precision. The constraint signs are assigned to equal numbers of positive and negative components*.

Two measurements were used to evaluate the quality of the our models. The overlap metric (Fitzgerald et al., 2011a):

(22) $$\begin{array}{l} O(\text{X},\text{Y})=\frac{\sqrt[{r}]{\left|\text{Det}\left(\text{X}{\text{Y}}^{\text{T}}\right)\right|}}{\sqrt[{2r}]{\left|\text{Det}\left(\text{X}{\text{X}}^{\text{T}}\right)\right|}\sqrt[{2r}]{\left|\text{Det}\left(\text{Y}{\text{Y}}^{\text{T}}\right)\right|}} \end{array}$$

measures how well the receptive field is recovered as measured on an interval O∈[0,1] where 0 means the two subspaces, **X**, **Y** (**X** and **Y** are generic matrices and unrelated to any other variables defined in the paper), are complementary while 1 means the subspaces span the same range space. The overlap metric allowed us to compare the quality of the *r*_opt_ recovered vectors of highest variance in **Ω** (Equation 11) to the model neuron subspace defined in the matrix **F**. Of course, since **F** was not available for the avian neurons, this measure was not used to evaluate solutions on the avian data. A second measure of the quality of the fit was the predictive power of the models in the reserved test sets. This was done by calculating the negative log-likelihood *L*_test_(*a*, **h**, **J**) evaluated over the test sets composed of the remaining data samples that were not used to train or cross-validate models (see Figure 4). In this latter assessment, models with minimal *L*_test_ were the best at predicting neural responses and assumed to recover better approximations of the underlying receptive field. Our application of these assessments to the model neurons were consistent with this assumption (Figure 1).

Once the mean components were recovered from **J**_sym_ averaged across jackknives, the FB method was applied to the avian data using the same data divisions as before (4 jackknives with 70%/20%/10% samples reserved for training, cross-validation, and testing). The FB basis set size was determined by finding the number of vectors, **c**_*k*_, necessary to saturate the negative log-likelihood up to a precision of ~ 10^−4^. Both logical AND and logical OR functions were fit for each neuron.

### 4.7. Resolving inconsistent optimal rank

In cases where model parameters are determined from multiple training and cross-validation sets, there is a risk that, due to the unique biases of each data set's sampling of stimulus and response space, the datasets may produce weights that disagree on the value of *r*_opt_, the optimal rank of **J**. For such cases, we use a statistical approach based in random matrix theory as a standard for deciding which eigenvalues of the mean 〈**J**_sym_〉 (Equation 11) across jackknives are significantly distinguishable from eigenvalues dominated by noise. The logic behind this statistical approach is as follows. Suppose that 〈**J**_sym_〉 is a large (*D* ≫ 1) matrix that comes from a distribution of random symmetric matrices $\hat{\text{J}}$ with elementwise mean 〈Ĵ_*i,j*_〉 = 0 and variance $\langle {Ĵ}_{i,j}^{2}\rangle ={\hat{\delta }}^{2}$. What is the probability that the *k*th eigenvalue of 〈**J**_sym_〉 comes from this distribution of random matrices?

According to the Wigner semi-circle law, in the limit *D* → ∞ the eigenvalues of random symmetric matrices of this type follow the probability distribution $P\left(\beta \right)=\frac{1}{2\pi {\hat{\delta }}^{2}}\sqrt{4{\hat{\delta }}^{2}-\beta^{2}}$ for $|\beta |\leq 4{\hat{\delta }}^{2}$ and 0 otherwise. In other words, the probability distribution is bounded at $-2|\hat{\delta }|\leq \beta \leq 2|\hat{\delta }|$ and β outside of these bounds is asymptotically improbable. Thus, if we can generate this probability distribution, we can define a principled method to find the mean optimal rank 〈*r*_opt_〉 using the bounds of the eigenvalue distribution, *P*(β), as a null hypothesis. Unfortunately, we do not know the probability distribution from which 〈**J**_sym_〉 is drawn so the analytic probability distribution is out of reach; but we can assume a conservative estimate of the bounds of the null hypothesis through an empirical estimate of a broad- ${\hat{\delta }}^{2}$ variance distribution. We make this empirical estimate by generating random symmetric matrices $\hat{\text{J}}$ where ${Ĵ}_{i,j}=\pm \langle J_{\text{sym}}^{\left(m,n\right)}\rangle$ and *m*, *n* are random integers on the interval [1, *D*]. The sign on Ĵ_*i,j*_ is chosen with equal probability to ensure that 〈Ĵ_*i,j*_〉 = 0 across the distribution while randomly drawing elements $\langle J_{\text{sym}}^{\left(m,n\right)}\rangle$ ensures a constant $\langle {Ĵ}_{i,j}^{2}\rangle$ across *i*, *j*. By aggregating the magnitude of the minimum and maximum eigenvalues from each of the random matrices, an estimate can be made on the bounds of the null hypothesis. With regard to this estimate of the bounds, *p*_*k*_ is defined as the probability that the magnitude of the *k*th eigenvalue of 〈**J**_sym_〉 is less than or equal to the magnitude of the bounds. If *p*_*k*_ < *p*_thres_ where *p*_thres_ ∈ [0, 1] is a significance threshold, then the *k*th eigenvalue of 〈**J**_sym_〉 is considered a statistically significant outlier from the null distribution with probability 1 − *p*. This estimate of the underlying probability distribution is conservative because it is designed to have a large variance and thus a large width for the semi-circle distribution such that an eigenvalue β of 〈**J**_sym_〉 is more likely to fall within the bounds of the null distribution. For a pseudocode outline of this algorithm, (see Algorithm 3).

**Algorithm 3:** Statistical approach for choosing 〈*r*_opt_〉

1:	**inputs: J**_sym_ ← 〈**J**_sym_〉 averaged across jackknives, *p*_thres_, the number of random matrices to generate *M*
2:	**initialization:** calculate the vector of eigenvalues **β** ← eig(**J**_sym_) arranged in descending order of magnitude, initialize empty vector **ζ** ← ∅, 〈*r*_opt_〉 ← 0
3:	
4:	**for** *m* = 1 to *M* **do**
5:	$\hat{\text{J}}\leftarrow$ *randomly sample D*(*D* + 1)/2 *elements of* ±**J**_sym_ *with uniform*
6:	*probability and generate a D* × *D symmetric matrix*.
7:	$\zeta \leftarrow \left[\zeta ,\;|\min\left(\text{eig}\left(\hat{\text{J}}\right)\right)|,\;\max\left(\text{eig}\left(\hat{\text{J}}\right)\right)\right]$ ⊳ Append magnitude of maximum and minimum eigenvalues of $\hat{\text{J}}$
8:	
9:	**for** *k* = 1 to *D* **do**
10:	$p\leftarrow \frac{1}{2M}\overset{2M}{\underset{m=1}{\sum }}H\left(\zeta_{m}-|\beta_{k}|\right)$ ⊳ *H*(·) is the Heaviside step function
11:	**if** *p* ≥ *p*_thres_ **then**
12:	**break**
13:	**else**
14:	〈*r*_opt_〉 ← *k*
15:	
16:	**output:** 〈*r*_opt_〉

### 4.8. Resources

The interior-point method, block coordinate descent algorithm, and FB method were written in Python 2.7 using standard numerical packages numpy (version 1.11.1) and scipy (version 0.18.1) and the machine learning package Theano (version 0.8.2) (Al-Rfou et al., 2016). These packages were installed through Anaconda (version 1.5.1) and were linked against Intel MKL (version 1.1.2) for CPU parallelization of linear algebra operations. Theano was chosen because it conveniently allows investigators to flexibly choose between using graphics processing units (GPUs) or central processing units (CPUs) as a backend to the optimization code without requiring modification to the code itself. We initially experimented with using GPUs to optimize the low-rank MNE models but found that the limitation to 32-bit floating-point precision on the available GPUs was inadequate without much hands-on tuning of the optimization parameters of the interior-point method which was not ideal for applications involving large datasets. In particular, using 32-bit floating-point precision in the algorithm often led to ill-conditioning of the Hessian matrix. These issues with 32-bit floating-point precision were replicated on CPUs as well. On the other hand, using 64-bit floating-point precision on CPUs did not present any issues with convergence or ill-conditioning. Consequently, we performed our low-rank MNE optimizations on a cluster of CPUs using 64-bit floating-point precision. The FB method, on the other hand, did not have any issues with precision so we ran these optimizations on GPUs using 32-bit precision. The optimization of full-rank and first-order MNE problems was done in C using OpenMP (version 4.0) and OpenBlas (version 0.2.14) for CPU parallelization. Figures were generated using MATLAB (version R2016b) and Inkscape (version 0.92.1).

## Ethics statement

This study was carried out with the approval of the Animal and Use Committee at University of California, Berkeley.

## Author contributions

The author contributions are itemized below: Model development-JK; Data acquisition-FT; Data analysis and interpretation-JK, TS; Drafting of the manuscript/revising for critically important intellectual content: JK, FT, TS.

### Conflict of interest statement

The authors declare that the research was conducted in the absence of any commercial or financial relationships that could be construed as a potential conflict of interest.
