# Supplementary material for: A Low-Rank Method for Characterizing High-Level Neural Computations
Source: Front Comput Neurosci. 2017 Jul 31;11:68. doi: 10.3389/fncom.2017.00068 (PMC5534486; doi:10.3389/fncom.2017.00068)
Supplement: Supplementary file 1 [file DataSheet1.pdf]

5 APPENDIX

5.1 Alternative constraint formulations

The advantage of the linear equality constraints (Eq 6) is that they provide strong optimality guarantees while scaling as a function of rD but come with the disadvantage of requiring the choice of π_k for each pair $\mathbf{U}_{\bullet,k}$ and $\mathbf{V}_{\bullet,k}$. There are several other possible equality constraints that satisfy $\mathrm{rank}(\mathbf{J}_{\mathrm{sym}}) = \mathrm{rank}(\mathbf{J})$ and do not require choosing π_k but they each are accompanied by their own disadvantages. A few possible alternatives are summarized below.

Two simple choices are the bilinear constraints $\mathbf{U}\mathbf{V}^{\mathrm{T}} = \mathbf{V}\mathbf{U}^{\mathrm{T}}$ which enforce symmetry and the quadratic constraints

$$\mathbf{U}\mathbf{U}^{\mathrm{T}} = \mathbf{V}\mathbf{V}^{\mathrm{T}}, \tag{23}$$

the latter of which can be proven to satisfy $\mathrm{rank}(\mathbf{J}_{\mathrm{sym}}) = \mathrm{rank}(\mathbf{J})$ by showing $\boldsymbol{\mathcal{P}}_{\boldsymbol{\mathcal{N}}}(\mathbf{U})\mathbf{V} = \mathbf{0}$ and $\boldsymbol{\mathcal{P}}_{\boldsymbol{\mathcal{N}}}(\mathbf{V})\mathbf{U} = \mathbf{0}$ (Fazel et al., 2003). The trouble with the bilinear and quadratic equality constraints is that they require optimizing $D(D-1)/2$ and $D(D+1)/2$ unique constraints making optimization with interior-point methods only tenable when D is small. Since the number of constraints does not depend on r, it means that block coordinate descent is not an option to decrease dimensionality in these cases. Furthermore, Hessian approximation techniques like L-BFGS usually do not attempt to approximate the Jacobian of the constraints and therefore will also not lead to a sufficient decrease in the dimensionality of the problem (Nocedal and Wright, 2006; Wächter and Biegler, 2006). With present memory and speed limitations, the best option in this case would be to use a constrained optimization technique that does not require second-order information; for instance, optimizing an augmented Lagrangian with conjugate gradient descent. In our experience, however, first-order methods may require more problem-specific tweaking of the optimization parameters (e.g. update parameters for the barrier function in an augmented Lagrangian method) to attain reasonable convergence speed compared to second-order methods. This may make first-order methods less attractive for large datasets.

Another possible alternative to the linear constraints is to instead insist that each $\mathbf{U}_{\bullet,k}$ and $\mathbf{V}_{\bullet,k}$ pair is parallel via the equality constraint

$$\left(\mathbf{U}_{\bullet,k}^{\mathrm{T}}\mathbf{V}_{\bullet,k}\right)^2 = \left\|\mathbf{U}_{\bullet,k}\right\|_2^2 \left\|\mathbf{V}_{\bullet,k}\right\|_2^2 \tag{24}$$

which comes from setting the square of the dot product of normalized $\mathbf{U}_{\bullet,k}$ and $\mathbf{V}_{\bullet,k}$ to 1. The advantages of this form for the equality constraints is that it scales as r and is thus lower dimensional than the linear equality constraints while not requiring a choice of π_k. Compared to the other constraints, however, this constraint is quite nonlinear as the Jacobians in the Hessian will depend explicitly on $\mathbf{U}$ and $\mathbf{V}$. This constraint may still behave well in practice, but it is not clear if convergence to a solution can be guaranteed. Indeed, experiments with this formulation often got stuck at non-stationary points which would appear to indicate that it does not behave well at least with interior-point methods. There are other variations one can try, including discontinuous variations of Eq 24 where the power-2 is replaced by the absolute value, but we will not explore these further.

Lastly, an alternative form of the constraints that may be attempted is a modification of the linear equality constraints (Eq 6) where the discretization of the π_k parameters is relaxed such that π_k can take on any real number. In this case, π_k would ensure that $\mathbf{U}_{\bullet,k}$ and $\mathbf{V}_{\bullet,k}$ are equal up to a signed scaling factor.

The downside of the relaxation is that one either needs to insert $\mathbf{U}_{\bullet,k} = -\pi_k \mathbf{V}_{\bullet,k}$ leading to a nonlinear optimization over a third-order polynomial or use the Lagrangian method in which case the Hessian of f (Eq 10) would be rank-deficient.

5.2 Optimality conditions

Here we show the conditions under which a stationary point of $\mathcal{L}$ (Eq 9) is a feasible local minimum of f (Eq 10) and derive some useful quantities for the subsequent discussion. In order to certify that a weight vector,

$$\mathbf{x}^{\mathrm{T}} = \left[\begin{array}{ccccc} a, & \mathbf{h}^{\mathrm{T}}, & \mathbf{Q}_{\bullet,1}^{\mathrm{T}}, & \cdots, & \mathbf{Q}_{\bullet,r}^{\mathrm{T}} \end{array} \right], \tag{25}$$

is a feasible local minimizer of $f(\mathbf{x})$, the first-order necessary conditions, also known as the Karush-Kuhn-Tucker (KKT) conditions, and the second-order sufficient conditions must be satisfied (Nocedal and Wright, 2006). The KKT conditions state that the gradient of $\mathcal{L}(\mathbf{x}, \mathbf{\Lambda})$ with respect to $\mathbf{x}$ is zero and $\mathbf{x}$ must be a feasible point in weight space. The second-order sufficient conditions require that the Hessian of $f(\mathbf{x})$ is positive semidefinite along feasible descent directions near $\mathbf{x}$.

The necessary conditions for $\mathbf{x}$ to be a feasible local minimizer of f appear in Prop 1 where ∇_a, $\boldsymbol{\nabla}_{\mathbf{h}}$, and $\boldsymbol{\nabla}_{\mathbf{Q}_{\bullet,k}}$ are the gradient operators with respect to a, $\mathbf{h}$, and $\mathbf{Q}_{\bullet,k}$, respectively, and

$$\mathbf{D}_t = \left[\begin{array}{cc} \mathbf{0}, & \mathbf{s}_t\mathbf{s}_t^{\mathrm{T}} \\ \mathbf{s}_t\mathbf{s}_t^{\mathrm{T}}, & \mathbf{0} \end{array} \right] \tag{26}$$

is a quadratic feature matrix.

PROPOSITION 1. *Karush-Kuhn-Tucker (KKT) conditions: the first-order necessary conditions for a feasible local minimum of $f(\mathbf{x})$ are*

$$\nabla_a \mathcal{L} = \frac{1}{N} \sum_t (P_t - y_t) = 0 \tag{27}$$

$$\boldsymbol{\nabla}_{\mathbf{h}} \mathcal{L} = \frac{1}{N} \sum_t (P_t - y_t)\mathbf{s}_t = \mathbf{0} \tag{28}$$

$$\boldsymbol{\nabla}_{\mathbf{Q}_{\bullet,k}} \mathcal{L} = \left[\frac{1}{N} \sum_t (P_t - y_t)\mathbf{D}_t + \epsilon_k \mathbf{I} \right] \mathbf{Q}_{\bullet,k} - \mathbf{A}_{k,k}^{\mathrm{T}} \mathbf{\Lambda}_{\bullet,k} = \mathbf{0} \textit{ for all } k \tag{29}$$

where $\mathbf{A}_{k,k}^{\mathrm{T}} = \boldsymbol{\nabla}_{\mathbf{Q}_{\bullet,k}} \mathbf{w}_k^{\mathrm{T}}$ (Eq 7) is the Jacobian of the constraint vector with respect to $\mathbf{Q}_{\bullet,k}$ and

$$\mathbf{w}_k = \mathbf{0} \textit{ for all } k \tag{30}$$

are the feasibility conditions.

With the constraints being linear and the Jacobian being full row-rank, all $\mathbf{w}_k$ satisfy the linear independence constraint qualification regularity condition and therefore the KKT conditions are guaranteed to be satisfied at a feasible stationary point of $\mathcal{L}$ (Nocedal and Wright, 2006). Notably, when the KKT conditions are satisfied $\mathbf{Q}_{\bullet,k}$ is complementary to (i.e. is a null space component of) Eq 29 because $\mathbf{Q}_{\bullet,k}^{\mathrm{T}}\mathbf{A}_{k,k}^{\mathrm{T}} = \mathbf{w}_k^{\mathrm{T}} = \mathbf{0}$

(Eq 8) and therefore

$$\mathbf{Q}_{\bullet,k}^{\mathrm{T}}\left[\frac{1}{N}\sum_t (P_t - y_t)\mathbf{D}_t + \epsilon_k \mathbf{I}\right]\mathbf{Q}_{\bullet,k} = 0 \Rightarrow \left[\frac{1}{N}\sum_t (P_t - y_t)\mathbf{D}_t + \epsilon_k \mathbf{I}\right]\mathbf{Q}_{\bullet,k} = \mathbf{0} \tag{31}$$

where the right-hand-side of the arrow follows from the fact that the bracketed term is a symmetric matrix. In other words, $\mathbf{Q}_{\bullet,k}$ is complementary to the quadratic gradient found in the bracketed term. This also implies that the Lagrange multipliers, $\mathbf{\Lambda}_{\bullet,k}$, are components of the null column-space of the partial Jacobian matrix, $\mathbf{A}_{k,k}$, for each k. This result will become important in the following discussion of locally/globally optimal regularization domains.

For nonconvex problems, the KKT conditions in Prop 1 are insufficient to guarantee that a stationary point is a feasible local minimum of f. Instead, we turn to the second-order sufficient conditions.

PROPOSITION 2. *Second-order sufficient conditions: if the KKT conditions in Prop 1 are satisfied at point* $\mathbf{x}^*$ *in weight space and*

$$\boldsymbol{\mathcal{S}} = \boldsymbol{\mathcal{N}}(\mathbf{A})\nabla^2_{\mathbf{xx}} f|_{\mathbf{x}^*}\boldsymbol{\mathcal{N}}(\mathbf{A})^{\mathrm{T}} \geq 0 \tag{32}$$

where $\mathbf{A}$ *is the full* $rD \times (1 + D + 2rD)$ *Jacobian matrix of the constraints and* $\boldsymbol{\mathcal{N}}(\mathbf{A})$ *returns the null space of* $\mathbf{A}$*, then* $\mathbf{x}^*$ *is a feasible local minimum of* f*.*

Intuitively, the second-order sufficient conditions mean that the Hessian must be positive semidefinite along feasible descent directions arbitrarily close to the stationary point $\mathbf{x}^*$.

In terms of z_t (Eq 1), the Hessian with respect to $\mathbf{x}$ may be written as

$$\nabla^2_{\mathbf{xx}} f = \underbrace{\frac{1}{N}\sum_t P_t(1 - P_t)(\nabla_{\mathbf{x}} z_t)(\nabla_{\mathbf{x}} z_t)^{\mathrm{T}}}_{\text{positive semidefinite, } \mathbf{RR}^{\mathrm{T}}} + \underbrace{\frac{1}{N}\sum_t (P_t - y_t)\nabla^2_{\mathbf{xx}} z_t}_{\text{indefinite, } \mathbf{M}} + \nabla^2_{\mathbf{xx}}\ell_* \tag{33}$$

where the Hessian operator is defined as $\nabla^2_{\mathbf{xx}} = \nabla_{\mathbf{x}}\nabla^{\mathrm{T}}_{\mathbf{x}}$,

$$\nabla^2_{\mathbf{xx}} z_t = \begin{bmatrix} \mathbf{0}, & \mathbf{0}, & \cdots, & \mathbf{0} \\ \mathbf{0}, & \mathbf{D}_t, & \ddots, & \vdots \\ \vdots, & \ddots, & \ddots, & \mathbf{0} \\ \mathbf{0}, & \cdots, & \mathbf{0}, & \mathbf{D}_t \end{bmatrix}, \tag{34}$$

is a symmetric block diagonal indefinite matrix and

$$\nabla^2_{\mathbf{xx}} \ell_* = \begin{bmatrix} \mathbf{0}, & \mathbf{0}, & \cdots, & \mathbf{0} \\ \mathbf{0}, & \epsilon_1\mathbf{I}, & \ddots, & \vdots \\ \vdots, & \ddots, & \ddots, & \mathbf{0} \\ \mathbf{0}, & \cdots, & \mathbf{0}, & \epsilon_r\mathbf{I} \end{bmatrix} \tag{35}$$

is a strictly positive semidefinite diagonal matrix. Due to the indefinite matrix, $\mathbf{M}$ (see definition in Eq 33), f is a nonconvex function.

5.3 Locally and globally optimal regularization domains

Generally speaking, the fact that the low-rank MNE optimization problem is nonconvex invites the possibility that there are multiple local minima, many of which may be suboptimal. However, since the nuclear-norm regularization penalty is convex, it can be shown that there is a regularization domain for which any solution to the low-rank MNE problem (Eq 8) is guaranteed to be globally optimal. We first observe that there is some value of the regularization parameters for which the Hessian of f (Eq 33) becomes positive semidefinite at a given $\mathbf{x}$.

PROPOSITION 3. *For a given* $\mathbf{x}$*, there is a threshold value of* ϵ_k *that satisfies* $\epsilon_k < \lambda_{\max}(\mathbf{M})$ *where* $\lambda_{\max}(\mathbf{M})$ *is the largest eigenvalue of* $\mathbf{M}$ *such that if all* ϵ_k *are greater than or equal to this threshold then* $\nabla^2_{\mathbf{xx}} f$ *evaluated at* $\mathbf{x}$ *is guaranteed to be positive semidefinite.*

PROOF. Under the assumption of fixed $\mathbf{x}$, the characteristic polynomial of $\mathbf{M} + \nabla^2_{\mathbf{xx}}\ell_*$ (Eq 33) is (using the block LDU decomposition)

$$\det\left(\mathbf{M} + \nabla^2_{\mathbf{xx}}\ell_* - \lambda\mathbf{I}\right) = -\lambda \prod_{k=1}^{r} \det\left(\left[\lambda - \epsilon_k\right]^2 \mathbf{I} - \left[\nabla_{\mathbf{J}} L\right]^2\right) = 0 \tag{36}$$

where λ is an eigenvalue of the Hessian and $\mathbf{I}$ is the identity matrix. For any λ that is a solution, there is a corresponding eigenvalue, $\lambda' = \epsilon_k \pm |\lambda - \epsilon_k|$, symmetric across ϵ_k that is also a solution. Since all $\epsilon_k \geq 0$, the minimum possible eigenvalue of $\mathbf{M} + \nabla^2_{\mathbf{xx}}\ell_*$ at a given $\mathbf{x}$ occurs when any $\epsilon_k = 0$ and is equal to the smallest eigenvalue of $\mathbf{M}$, $\lambda_{\min}(\mathbf{M}) \equiv -\lambda_{\max}(\mathbf{M})$. Therefore, if $\epsilon_k \geq \lambda_{\max}(\mathbf{M})$ for all k at a given $\mathbf{x}$, then the Hessian is guaranteed to be positive semidefinite at $\mathbf{x}$. Since $\lambda_{\min}(\nabla^2_{\mathbf{xx}} f) \geq \lambda_{\min}(\mathbf{R}\mathbf{R}^{\mathrm{T}}) + \lambda_{\min}(\mathbf{M} + \nabla^2_{\mathbf{xx}}\ell_*)$, $\epsilon_k = \lambda_{\max}(\mathbf{M})$ is an upper bound on the value of ϵ_k above which the Hessian becomes positive semidefinite for a given $\mathbf{x}$.

Now, suppose we design a (nonlinear) semidefinite program (SDP) equivalent to the low-rank MNE problem (Eq 8),

$$\min_{a,\mathbf{h},\mathbf{X}_1,\cdots,\mathbf{X}_r} f(a, \mathbf{h}, \mathbf{X}_1, \cdots, \mathbf{X}_r)$$
$$\text{subject to} \left\{ \begin{array}{c} \mathbf{J}_k - \mathbf{J}_k^{\mathrm{T}} = \mathbf{0}, \\ \mathrm{rank}(\mathbf{X}_k) \leq 1, \\ \mathbf{X}_k \geq 0 \end{array} \right\} \text{for all k} \tag{37}$$

where $\mathbf{X}_k \equiv \mathbf{Q}_{\bullet,k}\mathbf{Q}_{\bullet,k}^{\mathrm{T}}$ is a $2D \times 2D$ rank-one matrix with regularization parameter ϵ_k, $\mathbf{J}_k$ is the upper-right $D \times D$ block of $\mathbf{X}_k$, and $\mathbf{X}_k \geq 0$ indicates $\mathbf{X}_k$ is constrained to be positive semidefinite. In this form, the SDP is nonconvex with potentially many local minima. If, however, we relax the SDP by eliminating the rank constraint (or equivalently modifying it to $\mathrm{rank}(\mathbf{X}_k) \leq 2D$ for all k), then the problem becomes convex.

PROPOSITION 4. *(based on proposition 4 from Bach et al. (2008) and theorem 2 from Haeffele et al. (2014)) If* $\mathbf{x}^*$ *is a feasible local minimizer of the equivalent SDP (Eq 37) and*

$$\min(\epsilon_1, \cdots, \epsilon_r) \geq \lambda_{\max}\left(\frac{1}{N}\sum_t (P_t - y_t)\mathbf{D}_t\right) = 2\lambda_{\max}(\nabla_{\mathbf{X}_k} L) \textit{ for any } k \tag{38}$$

solved at $\mathbf{x}^*$ then $\mathbf{x}^*$ is a feasible global minimizer of the equivalent SDP (note that $\nabla_{\mathbf{X}_k} L$ is a $2D \times 2D$ gradient matrix instead of a gradient vector).

PROOF. According to Prop 3, there is some value for each ϵ_k above which the eigenvalue spectrum of $2\nabla_{\mathbf{X}_k} f$ is strictly positive for a given $\mathbf{x}$. However, since $\nabla_{\mathbf{X}_j} L = \nabla_{\mathbf{X}_k} L$ for any j and k, the threshold value of ϵ_k such that the Hessian is positive semidefinite is the same for all k. Thus, if the minimal assigned value of ϵ_k across all k satisfies Eq 38, the Hessian is positive semidefinite at $\mathbf{x}$. If $\mathbf{x}^*$ is a solution to the low-rank MNE minimization problem (Eq 8) and Prop 4 is true, then all $\nabla_{\mathbf{X}_k} f$ are positive semidefinite matrices and $\nabla_{\mathbf{X}_k} f \mathbf{X}_k^* = \mathbf{0}$ (Eq 31) at $\mathbf{x}^*$. It follows that $\mathbf{x}^*$ is then a solution to the relaxed SDP and a global minimizer of f (Burer and Monteiro, 2003; Bach et al., 2008; Haeffele et al., 2014). This is true because the corresponding SDP weights for a, $\mathbf{h}$, and all $\mathbf{X}_k$ are a feasible local minimizer of the relaxed SDP along feasible descent directions shown via the first-order Taylor series expansion of f with respect to all $\mathbf{X}_k$ about solution a^*, $\mathbf{h}^*$, $\mathbf{X}^* = \sum_k \mathbf{X}_k^*$:

$$\begin{aligned} f(a^*, \mathbf{h}^*, \mathbf{X}) &\approx f(a^*, \mathbf{h}^*, \mathbf{X}^*) + \sum_k \mathrm{Tr}\left(\left[\nabla_{\mathbf{X}_k} f(a^*, \mathbf{h}^*, \mathbf{X}^*)\right]^{\mathrm{T}} \left[\mathbf{X}_k - \mathbf{X}_k^*\right]\right) \\ &= f(a^*, \mathbf{h}^*, \mathbf{X}^*) + \sum_k \mathrm{Tr}\left(\left[\nabla_{\mathbf{X}_k} f(a^*, \mathbf{h}^*, \mathbf{X}^*)\right]^{\mathrm{T}} \mathbf{X}_k\right). \end{aligned} \tag{39}$$

No feasible $\mathbf{X}_k$ can locally decrease f because the trace of a product of two positive semidefinite matrices is greater than or equal to zero. Furthermore, since f is a convex function of a, $\mathbf{h}$, and $\mathbf{X}_k$, this local information is sufficient to conclude that $\mathbf{x}^*$ is at a feasible global minimum of f. Therefore, solutions to the low-rank MNE minimization problem (Eq 8) and the rank-constrained SDP (Eq 37) are globally optimal for a given rank and set of regularization parameters provided Prop 4 is satisfied.

Low-rank MNE models optimized to satisfy Prop 4 constitute a regularization domain of globally optimal solutions to the low-rank MNE minimization problem. Low-rank models within this globally optimal domain can be a consistent, good approximation to $\mathbf{J}$ of a given maximum rank r provided the training data is sufficiently representative of the underlying ground truth value of $\mathbf{J}$. This can be particularly helpful when attempting to extract low-rank $\mathbf{J}$ from exceptionally high-dimensional problems that are impractical to solve at full-rank.

A secondary consequence of the above analysis is that, in the locally optimal regularization domain, solutions are unique along a given unit matrix, $\hat{\mathbf{Q}} = \frac{\mathbf{Q}}{\|\mathbf{Q}\|_{\mathrm{F}}}$, where $\|\cdot\|_{\mathrm{F}}$ is the Frobenius norm.

PROPOSITION 5. *Given a solution $\mathbf{x}^*$ to the low-rank MNE problem, the associated quadratic weights, $\mathbf{Q}^*$, are a unique solution along the unit matrix $\hat{\mathbf{Q}}^*$ up to a change in sign of the columns (i.e. $\pm\mathbf{Q}^*_{\bullet,k}$ are equivalent solutions).*

PROOF. Unlike in the globally optimal regularization domain where $\nabla_{\mathbf{X}_k} f$ is a positive semidefinite matrix at solution $\mathbf{x}^*$, in the locally optimal regularization domain $\nabla_{\mathbf{X}_k} f$ may be indefinite at a solution $\mathbf{x}^*$. However, f is still a convex function of the SDP weights, a, $\mathbf{h}$, and $\mathbf{X}_k$. Furthermore, $\mathbf{X}_k^*$ is still complementary to $\nabla_{\mathbf{X}_k} f$ (Eq 39) at the solution. Therefore, $\mathbf{X}_k^*$ is a unique solution along unit matrix $\hat{\mathbf{X}}_k^*$. This unit direction matrix is represented in factorized space by $\hat{\mathbf{X}}_k^* = (\pm\hat{\mathbf{Q}}^*_{\bullet,k})(\pm\hat{\mathbf{Q}}^{*\mathrm{T}}_{\bullet,k}) = \hat{\mathbf{Q}}^*_{\bullet,k}\hat{\mathbf{Q}}^{*\mathrm{T}}_{\bullet,k}$. This factorization of the unit matrix, $\hat{\mathbf{X}}_k^*$, defines a unique direction in factorized space, $\hat{\mathbf{Q}}^*_{\bullet,k}$, and thus $\mathbf{Q}^*_{\bullet,k}$ from solution $\mathbf{x}^*$ is also a unique solution up to a change in sign.

Intuitively, this means that a local minimum constrained to the unit direction $\hat{\mathbf{Q}}$ is a global minimum along $\hat{\mathbf{Q}}$. In the case of degeneracy in $\mathbf{Q}$, arbitrary rotations of degenerate vectors are globally optimal along any unit direction in this degenerate subspace. This result does not find use in this paper but we provide it here for completeness in case future advancements in numerical optimization techniques are able to exploit this structure to efficiently obtain certifiable global minima of problems in the locally optimal domain. At the moment, this property would theoretically decrease the number of iterations necessary to reach the global minimum using a branch and bound algorithm but the algorithm would still be at worst EXPTIME and impractical to implement for large D.

To investigate the existence of suboptimal local minima in the locally optimal domain, we took an empirical approach where we searched for a counterexample to the hypothesis that, despite nonconvexity, there are no suboptimal local minima. This was done by drawing random weights, $\mathbf{x}$, from a normal distribution with $D = 2$ and $r_{\text{opt}} = 2$. For each problem, $N = 100$ stimulus feature vectors were also drawn from a normal distribution. The response was generated using Eq 1. Then $r = 1$ low-rank MNE models were fit 10 times for each randomly drawn problem with different random initializations of the weights. If two models for a given random problem differed in negative log-likelihood by $1 \cdot 10^{-4}$ at their respective minima, then that problem qualified as a potential counterexample and was stored. To ensure that the difference in negative log-likelihood was not due to imprecise fitting, the detected counterexamples were verified by plotting f (Eq 10) in $\mathbf{U}$ space and observing the existence of spatially separated minima. We found that there are indeed counterexamples to the hypothesis and it is therefore possible that suboptimal local minima will exist in a given problem. However, we remark that suboptimal local minima are seemingly rare among problems drawn from a normal distribution, often requiring the generation of on the order of $\sim 10^3$ random problems to find one with a suboptimal local minimum.

5.4 Convergence of the block coordinate descent algorithm

Since the interior-point algorithm (Nocedal and Wright, 2006) used to solve each block problem already guarantees global convergence to a feasible local minimizer of a given block of weights, $\mathbf{x}_k$, with fixed regularization parameter, ϵ_k, each cycle through the r blocks (Eq 13) of the block coordinate descent algorithm leads to a monotonically decreasing series $f(\mathbf{x}_1^{(j)}) \geq f(\mathbf{x}_2^{(j)}) \geq ... \geq f(\mathbf{x}_r^{(j)})$ for the jth cycle. Because $f \geq 0$ is bounded from below on the domain of the weights, the cost function value cannot decrease indefinitely and the series saturates as $j \to \infty$ to a stationary point. At this stationary point, the gradient of the Lagrangian with respect to each block is simultaneously zero and all $\boldsymbol{\nabla}^2_{\mathbf{x}_k\mathbf{x}_k} f$ are simultaneously positive semidefinite when projected into the null space of the constraints,

$$\boldsymbol{\mathcal{S}}_k = \boldsymbol{\mathcal{N}}(\mathrm{A}^{(k)}) \boldsymbol{\nabla}^2_{\mathbf{x}_k\mathbf{x}_k} f|_{\mathbf{x}_k^*} \boldsymbol{\mathcal{N}}(\mathrm{A}^{(k)})^{\mathrm{T}} \geq 0. \tag{40}$$

However, satisfying these conditions alone does not guarantee that this stationary point is a feasible local minimizer of the low-rank MNE problem (Eq 8). It can be shown that this stationary point is, in fact, a feasible local minimum of $\mathcal{L}$ by verifying that the necessary and sufficient conditions of the full problem (Prop 1 & 2 in appendix) are satisfied when the block KKT conditions (right-hand-side of Eq 14) and block second-order sufficient conditions (Eq 40) are satisfied.

In the following discussion, we will assume that the data has been prepared sufficiently according to Assumption 1, which may be done without loss of generality. We formally state the necessary and sufficient conditions under which a stationary point of the block coordinate descent is a feasible local minimizer of the low-rank MNE problem (Eq 8) in Prop 6.

ASSUMPTION 1. *The feature space satisfies*

$$\text{rank}\left(\frac{1}{N}\sum_t \begin{bmatrix} 1 \\ \mathbf{s}_t \end{bmatrix} \begin{bmatrix} 1, & \mathbf{s}_t^{\mathrm{T}} \end{bmatrix}\right) = D + 1 \tag{41}$$

or has been transformed in such a way that this is true.

Intuitively, this assumption means that the covariance of the stimulus feature space must be full-rank. If the stimulus space does not satisfy this assumption, one can, for example, project the stimuli into the subset of principal components with non-zero variance without loss of generality.

PROPOSITION 6. *If, for all k, $\mathbf{x}_k$ are feasible local minima of the block subproblems (Eq 13) where the block KKT conditions*

$$\boldsymbol{\nabla}_{\mathbf{x}_k}\boldsymbol{\mathcal{L}} = \mathbf{0}, \ \mathbf{w}_k = \mathbf{0} \tag{42}$$

and block second-order sufficient conditions $\boldsymbol{\mathcal{S}}_k \geq 0$ (Eq 40) are satisfied, then the equivalent weight vector, $\mathbf{x}$, of the full problem is a feasible local minimizer of the low-rank MNE problem (Eq 8).

PROOF. (Part 1: necessary conditions) The KKT conditions in Eq 27, 28, & 29 are trivially satisfied when the block gradients are zero. Also, each constraint vector $\mathbf{w}_k$ is only dependent on the kth block and therefore the feasibility conditions in Eq 30 are satisfied as well.

Showing that the second-order sufficient conditions are satisfied is a bit more involved. To make the proof less cumbersome, we define the abbreviations

$$\mathbf{A}^{*\mathrm{T}}_{i:j,i:j} = \begin{bmatrix} \mathbf{A}^{*\mathrm{T}}_{i,i}, & \mathbf{0}, & \cdots, & \mathbf{0} \\ \mathbf{0}, & \mathbf{A}^{*\mathrm{T}}_{i+1,i+1}, & \ddots, & \vdots \\ \vdots, & \ddots, & \ddots, & \mathbf{0} \\ \mathbf{0}, & \ldots, & \mathbf{0}, & \mathbf{A}^{*\mathrm{T}}_{j,j} \end{bmatrix}, \tag{43}$$

$$\mathbf{B} = \frac{1}{N}\sum_t P_t(1-P_t) \begin{bmatrix} 1 \\ \mathbf{s}_t \end{bmatrix} \begin{bmatrix} 1, & \mathbf{s}_t \end{bmatrix}, \tag{44}$$

$$\mathbf{R}_{i:j,\bullet}\mathbf{R}^{\mathrm{T}}_{i':j',\bullet} = \frac{1}{N}\sum_t P_t(1-P_t) \begin{bmatrix} \mathbf{D}_t\mathbf{Q}_{\bullet,i} \\ \mathbf{D}_t\mathbf{Q}_{\bullet,i+1} \\ \vdots \\ \mathbf{D}_t\mathbf{Q}_{\bullet,j} \end{bmatrix} \begin{bmatrix} \mathbf{D}_t\mathbf{Q}_{\bullet,i'} \\ \mathbf{D}_t\mathbf{Q}_{\bullet,i'+1} \\ \vdots \\ \mathbf{D}_t\mathbf{Q}_{\bullet,j'} \end{bmatrix}^{\mathrm{T}}, \tag{45}$$

$$\mathbf{Y}_{i:j,\bullet} = \frac{1}{N}\sum_t P_t(1-P_t) \begin{bmatrix} \mathbf{D}_t\mathbf{Q}_{\bullet,i} \\ \mathbf{D}_t\mathbf{Q}_{\bullet,i+1} \\ \vdots \\ \mathbf{D}_t\mathbf{Q}_{\bullet,j} \end{bmatrix} \begin{bmatrix} 1, & \mathbf{s}_t^{\mathrm{T}} \end{bmatrix}, \tag{46}$$

$$\mathbf{Z}_{i:j,i:j} = \frac{1}{N}\sum_t (P_t - y_t)\begin{bmatrix} \mathbf{D}_t, & \mathbf{0}, & \cdots, & \mathbf{0} \\ \mathbf{0}, & \mathbf{D}_t, & \ddots, & \vdots \\ \vdots, & \ddots, & \ddots, & \mathbf{0} \\ \mathbf{0}, & \cdots, & \mathbf{0}, & \mathbf{D}_t \end{bmatrix} + \begin{bmatrix} \epsilon_i\mathbf{I}, & \mathbf{0}, & \cdots, & \mathbf{0} \\ \mathbf{0}, & \epsilon_{i+1}\mathbf{I}, & \ddots, & \vdots \\ \vdots, & \ddots, & \ddots, & \mathbf{0} \\ \mathbf{0}, & \cdots, & \mathbf{0}, & \epsilon_j\mathbf{I} \end{bmatrix} \tag{47}$$

for $0 < i \le j \le r$ where i and j can be thought of as slicing indices (the same conditions apply to i' and j') that define a submatrix of a larger matrix. Under Assumption 1, $\mathbf{R}_{i:j,\bullet}\mathbf{R}_{i:j,\bullet}^{\mathrm{T}}$ is positive definite provided all $\mathbf{Q}_{\bullet,i}, \cdots, \mathbf{Q}_{\bullet,j} \neq \mathbf{0}$. If any $\mathbf{Q}_{\bullet,k} = \mathbf{0}$ for $i \le k \le j$, then $\mathbf{R}_{i:j,\bullet}\mathbf{R}_{i:j,\bullet}^{\mathrm{T}}$ is strictly positive semidefinite. The matrix $\mathbf{Z}_{i,j}$ is generally an indefinite matrix and has rank-deficiency $\operatorname{rank}\boldsymbol{\mathcal{N}}(\mathbf{Z}_{i,j}) \ge \operatorname{rank}\left([\mathbf{Q}_{\bullet,i}, \mathbf{Q}_{\bullet,i+1}, \cdots, \mathbf{Q}_{\bullet,j}]\right)$ (recall from Eq 31 that $\mathbf{Q}_{\bullet,k}$ is complementary to $\mathbf{Z}_{k,k}$). The matrix $\mathbf{B}$ is always positive definite.

In terms of these abbreviations, we rewrite $\boldsymbol{\mathcal{S}}_k$ (Eq 40) as

$$\begin{aligned} &\begin{bmatrix} \mathbf{I}, & \mathbf{0} \\ \mathbf{0}, & \mathbf{A}_{k,k}^{*} \end{bmatrix}\begin{bmatrix} \mathbf{B}, & \mathbf{Y}_{k,\bullet}^{\mathrm{T}} \\ \mathbf{Y}_{k,\bullet}, & \mathbf{R}_{k,\bullet}\mathbf{R}_{k,\bullet}^{\mathrm{T}} + \mathbf{Z}_{k,k} \end{bmatrix}\begin{bmatrix} \mathbf{I}, & \mathbf{0} \\ \mathbf{0}, & \mathbf{A}_{k,k}^{*\mathrm{T}} \end{bmatrix} \\ &= \begin{bmatrix} \mathbf{B}, & \mathbf{Y}_{k,\bullet}^{\mathrm{T}}\mathbf{A}_{k,k}^{*\mathrm{T}} \\ \mathbf{A}_{k,k}^{*}\mathbf{Y}_{k,\bullet}, & \mathbf{A}_{k,k}^{*}\left(\mathbf{R}_{k,\bullet}\mathbf{R}_{k,\bullet}^{\mathrm{T}} + \mathbf{Z}_{k,k}\right)\mathbf{A}_{k,k}^{*\mathrm{T}} \end{bmatrix} \end{aligned} \tag{48}$$

and take the Shur complement over $\mathbf{B}$

$$(\boldsymbol{\mathcal{S}}_k/\mathbf{B}) = \mathbf{A}_{k,k}^{*}\left(\mathbf{R}_{k,\bullet}\mathbf{R}_{k,\bullet}^{\mathrm{T}} + \mathbf{Z}_{k,k}\right)\mathbf{A}_{k,k}^{*\mathrm{T}} - \mathbf{A}_{k,k}^{*}\mathbf{Y}_{k,\bullet}\mathbf{B}^{-1}\mathbf{Y}_{k,\bullet}^{\mathrm{T}}\mathbf{A}_{k,k}^{*\mathrm{T}} \tag{49}$$

which is positive semidefinite and therefore

$$\boldsymbol{\Theta}_{k,k} = \mathbf{A}_{k,k}^{*}\left(\mathbf{R}_{k,\bullet}\mathbf{R}_{k,\bullet}^{\mathrm{T}} + \mathbf{Z}_{k,k}\right)\mathbf{A}_{k,k}^{*\mathrm{T}} \tag{50}$$

is positive semidefinite. Provided $\mathbf{Q}_{\bullet,k} \neq \mathbf{0}$, it is much more likely that $\boldsymbol{\Theta}_{k,k}$ will be positive definite than strictly positive semidefinite given the structural dissimilarity of $\mathbf{R}_{k,\bullet}\mathbf{R}_{k,\bullet}^{\mathrm{T}}$ and $\mathbf{Z}_{k,k}$. Even if $\boldsymbol{\Theta}_{k,k}$ is singular, an infinitesimal increase to ϵ_k would theoretically produce a full-rank approximation to $\boldsymbol{\Theta}_{k,k}$ with negligible impact on the weights. From here forward, it is assumed that $\boldsymbol{\Theta}_{k,k}$ is positive definite unless $\mathbf{Q}_{\bullet,k} = \mathbf{0}$.

We will now use the result in Eq 50 (and the surrounding discussion) and recursive application of the Schur complement to show that the second-order sufficient conditions (Prop 2) of the full problem are satisfied.

PROOF. (Part 2: sufficient conditions) The second-order sufficient conditions of the low-rank MNE problem (Prop 2) are rewritten in terms of Eq 43, 44, 45, 46, & 47

$$\begin{aligned} &\begin{bmatrix} \mathbf{I}, & \mathbf{0} \\ \mathbf{0}, & \mathbf{A}_{1:r,1:r}^{*} \end{bmatrix}\begin{bmatrix} \mathbf{B}, & \mathbf{Y}_{1:r,\bullet}^{\mathrm{T}} \\ \mathbf{Y}_{1:r,\bullet}, & \mathbf{R}_{1:r,\bullet}\mathbf{R}_{1:r,\bullet}^{\mathrm{T}} + \mathbf{Z}_{1:r,1:r} \end{bmatrix}\begin{bmatrix} \mathbf{I}, & \mathbf{0} \\ \mathbf{0}, & \mathbf{A}_{1:r,1:r}^{*\mathrm{T}} \end{bmatrix} \\ &= \begin{bmatrix} \mathbf{B}, & \mathbf{Y}_{1:r,\bullet}^{\mathrm{T}}\mathbf{A}_{1:r,1:r}^{*\mathrm{T}} \\ \mathbf{A}_{1:r,1:r}^{*}\mathbf{Y}_{1:r,\bullet}, & \mathbf{A}_{1:r,1:r}^{*}\left(\mathbf{R}_{1:r,\bullet}\mathbf{R}_{1:r,\bullet}^{\mathrm{T}} + \mathbf{Z}_{1:r,1:r}\right)\mathbf{A}_{1:r,1:r}^{*\mathrm{T}} \end{bmatrix}, \end{aligned} \tag{51}$$

Then one can take the Schur complement over $\mathbf{B}$

$$(\boldsymbol{\mathcal{S}}/\mathbf{B}) = \mathbf{\Theta}_{1:r,1:r} - \mathbf{A}^{*}_{1:r,1:r}\mathbf{Y}_{1:r,\bullet}\mathbf{B}^{-1}\mathbf{Y}^{\mathrm{T}}_{1:r,\bullet}\mathbf{A}^{*\mathrm{T}}_{1:r,1:r}, \tag{52}$$

where $\mathbf{\Theta}_{i:j,i':j'} = \mathbf{A}^{*}_{i:j,i:j}\left(\mathbf{R}_{i:j,\bullet}\mathbf{R}^{\mathrm{T}}_{i':j',\bullet} + \mathbf{Z}_{i:j,i':j'}\right)\mathbf{A}^{*\mathrm{T}}_{i':j',i':j'}$ and then recursively take the Schur complement of $\mathbf{\Theta}_{k:r,k:r}$ with respect to $\mathbf{\Theta}_{k,k}$ forming the sequence

$$\begin{aligned}
(\mathbf{\Theta}_{1:r,1:r}/\mathbf{\Theta}_{1,1}) &= \mathbf{\Theta}_{2:r,2:r} - \mathbf{\Theta}_{2:r,1}\mathbf{\Theta}^{-1}_{1,1}\mathbf{\Theta}_{1,2:r} \\
(\mathbf{\Theta}_{2:r,2:r}/\mathbf{\Theta}_{2,2}) &= \mathbf{\Theta}_{3:r,3:r} - \mathbf{\Theta}_{3:r,2}\mathbf{\Theta}^{-1}_{2,2}\mathbf{\Theta}_{2,3:r} \\
(\mathbf{\Theta}_{3:r,3:r}/\mathbf{\Theta}_{3,3}) &= \mathbf{\Theta}_{4:r,4:r} - \mathbf{\Theta}_{4:r,3}\mathbf{\Theta}^{-1}_{3,3}\mathbf{\Theta}_{3,4:r} \\
&\vdots \\
(\mathbf{\Theta}_{r-1:r,r-1:r}/\mathbf{\Theta}_{r-1,r-1}) &= \mathbf{\Theta}_{r,r} - \mathbf{\Theta}_{r-1:r,r-1}\mathbf{\Theta}^{-1}_{r-1,r-1}\mathbf{\Theta}_{r-1,r-1:r}.
\end{aligned} \tag{53}$$

If all $\mathbf{Q}_{\bullet,k} \neq \mathbf{0}$, it is apparent that the last equation in the sequence is positive definite because $\mathbf{\Theta}_{r-1,r-1}$ and $\mathbf{\Theta}_{r,r}$ are in block form (Eq 50) and each of the blocks are already known to be positive definite. We can then work backwards inserting the result of the equation below into the equation above, showing that each equation is positive definite, until we reach Eq 52 which will also be positive definite. Therefore, $\boldsymbol{\mathcal{S}}$ is positive definite and $\mathbf{x}$ is a feasible local minimum. Now suppose that $\mathbf{Q}$ is rank-deficient with rank $r^* < r$; then (without loss of generality) we can assume the first $r - r^*$ columns are zeros ($\mathbf{Q}_{\bullet,k} = \mathbf{0}$ for $k \leq r - r^*$). The $k \leq r - r^*$ rows and columns of $\mathbf{R}_{1:r,\bullet}\mathbf{R}^{\mathrm{T}}_{1:r,\bullet}$ are all zeros in this case and therefore the submatrix $\mathbf{\Theta}_{1:r-r^*,1:r-r^*} = \mathbf{Z}_{1:r-r^*,1:r-r^*}$ is block diagonal, the submatrix $\mathbf{\Theta}_{r-r^*+1:r,r-r^*+1:r}$ is positive definite, and the remaining submatrices all zeros. Since each $\mathbf{\Theta}_{k,k}$ for $k \leq r - r^*$ is singular, the inverses $\mathbf{\Theta}^{-1}_{k,k}$ in the first $r - r^*$ lines of the recursive Shur complement sequence (Eq 53) need to be replaced with the generalized inverse $\mathbf{\Theta}^{\dagger}_{k,k}$ and must satisfy the additional condition that $\boldsymbol{\mathcal{N}}(\mathbf{\Theta}_{k,k+1:r}) = \boldsymbol{\mathcal{N}}(\mathbf{A}^{*}_{k,k}\mathbf{R}_{k,\bullet}\mathbf{R}^{\mathrm{T}}_{k+1:r,\bullet}\mathbf{A}^{*\mathrm{T}}_{k+1:r,k+1:r})$ is a superset of $\boldsymbol{\mathcal{N}}(\mathbf{\Theta}_{k,k})$ for $\mathbf{\Theta}_{k:r,k:r}$ to be positive semidefinite (see the generalized Schur complement for background). The null space condition can be tested with the null space projection operator of $\mathbf{\Theta}_{k,k}$ acting on the row space of $\mathbf{\Theta}_{k,k+1:r}$

$$\boldsymbol{\mathcal{P}}_{\boldsymbol{\mathcal{N}}}(\mathbf{\Theta}_{k,k})\mathbf{\Theta}_{k,k+1:r} = \mathbf{0} \tag{54}$$

where the equality is true because $\mathbf{R}_{k,\bullet} = \mathbf{0}$ when $\mathbf{Q}_{\bullet,k} = \mathbf{0}$. Working backwards from the bottom of the sequence again, we see that $\boldsymbol{\mathcal{S}}$ must be positive semidefinite. Therefore, we conclude that the block coordinate descent algorithm converges to a feasible local minimizer of f consistent with Prop 6.

Note that the above analysis is equivalent to showing that block coordinate descent will converge when the constraints are directly substituted by setting $\mathbf{V}_{\bullet,k} = -\pi_k\mathbf{U}_{\bullet,k}$ for each k. This is true because $\boldsymbol{\mathcal{S}} \equiv \boldsymbol{\mathcal{S}}^{(\mathrm{sub})}$ (Eq 32) and $\boldsymbol{\mathcal{S}}_k \equiv \boldsymbol{\mathcal{S}}^{(\mathrm{sub})}_k$ (Eq 40) where $\boldsymbol{\mathcal{S}}^{(\mathrm{sub})}$ and $\boldsymbol{\mathcal{S}}^{(\mathrm{sub})}_k$ are the full and block second-order sufficient conditions, respectively, of the low-rank MNE problem with the constraints directly substituted.
